# Synergistic co-regulation and competition by a SOX9-GLI-FOXA phasic transcriptional network coordinate chondrocyte differentiation transitions

**DOI:** 10.1371/journal.pgen.1007346

**Published:** 2018-04-16

**Authors:** Zhijia Tan, Ben Niu, Kwok Yeung Tsang, Ian G. Melhado, Shinsuke Ohba, Xinjun He, Yongheng Huang, Cheng Wang, Andrew P. McMahon, Ralf Jauch, Danny Chan, Michael Q. Zhang, Kathryn S. E. Cheah

**Affiliations:** 1 School of Biomedical Sciences, LKS Faculty of Medicine, the University of Hong Kong, Pokfulam, Hong Kong; 2 Department of Stem Cell Biology and Regenerative Medicine, Eli and Edythe Broad-CIRM Center for Regenerative Medicine and Stem Cell Research, W.M. Keck School of Medicine of the University of Southern California, Los Angeles, California, United States of America; 3 Genome Regulation Laboratory, Guangzhou Institutes of Biomedicine and Health, Guangzhou, China; 4 Department of Biological Sciences, Center for Systems Biology, The University of Texas at Dallas, Dallas, Texas, United States of America; 5 MOE Key Laboratory of Bioinformatics, Center for Synthetic and Systems Biology, TNLIST, Tsinghua University, Beijing, China; Stanford University School of Medicine, UNITED STATES

## Abstract

The growth plate mediates bone growth where SOX9 and GLI factors control chondrocyte proliferation, differentiation and entry into hypertrophy. FOXA factors regulate hypertrophic chondrocyte maturation. How these factors integrate into a Gene Regulatory Network (GRN) controlling these differentiation transitions is incompletely understood. We adopted a genome-wide whole tissue approach to establish a **G**rowth **P**late **D**ifferential **G**ene **E**xpression **L**ibrary (GP-DGEL) for fractionated proliferating, pre-hypertrophic, early and late hypertrophic chondrocytes, as an overarching resource for discovery of pathways and disease candidates. *De novo* motif discovery revealed the enrichment of SOX9 and GLI binding sites in the genes preferentially expressed in proliferating and prehypertrophic chondrocytes, suggesting the potential cooperation between SOX9 and GLI proteins. We integrated the analyses of the transcriptome, SOX9, GLI1 and GLI3 ChIP-seq datasets, with functional validation by transactivation assays and mouse mutants. We identified new SOX9 targets and showed SOX9-GLI directly and cooperatively regulate many genes such as *Trps1*, *Sox9*, *Sox5*, *Sox6*, *Col2a1*, *Ptch1*, *Gli1* and *Gli2*. Further, FOXA2 competes with SOX9 for the transactivation of target genes. The data support a model of SOX9-GLI-FOXA phasic GRN in chondrocyte development. Together, SOX9-GLI auto-regulate and cooperate to activate and repress genes in proliferating chondrocytes. Upon hypertrophy, FOXA competes with SOX9, and control toward terminal differentiation passes to FOXA, RUNX, AP1 and MEF2 factors.

## Author summary

In the development of the mammalian growth plate, while several transcription factors are individually well known for their key roles in regulating phases of chondrocyte differentiation, there is little information on how they interact and cooperate with each other. We took an unbiased genome wide approach to identify the transcription factors and signaling pathways that play dominant roles in the chondrocyte differentiation cascade. We developed a searchable library of differentially expressed genes, GP-DGEL, which has fine spatial resolution and global transcriptomic coverage for discovery of processes, pathways and disease candidates. Our work identifies a novel regulatory mechanism that integrates the action of three transcription factors, SOX9, GLI and FOXA. SOX9-GLI auto-regulate and cooperate to activate and repress genes in proliferating chondrocytes. Upon entry into prehypertrophy, FOXA competes with SOX9, and control of hypertrophy passes to FOXA, RUNX, AP1 and MEF2 factors.

## Introduction

In the formation and longitudinal growth of endochondral bones, committed mesenchymal cells condense and differentiate into chondrocytes to form a growth plate, within which chondrocytes undergo coordinated and sequential differentiation phases of proliferation, cell cycle exit and hypertrophy, resulting in longitudinal bone growth[1, 2]. Endochondral bone formation requires tightly controlled proportions of the different chondrocyte populations, recognized by their distinct morphology, characteristic gene expression patterns, and organization into different zones. Firstly, round chondrocytes become proliferative, flattening to form columns. As proliferating chondrocytes (PCs) mature, they exit the cell cycle and enter a prehypertrophic phase. This phase is an important transition, which produces signals for maintaining proliferation on the one hand and on the other, to regulate the progression from proliferation to cell cycle exit, entry into a prehypertrophic state, followed by the final stages of differentiation in which the cells enlarge to form hypertrophic chondrocytes (HCs) and then become osteoblasts [1–4]. Disruption of the progression from one differentiation state to the next and the relative proportions results in skeletal defects such as chondrodysplasia [5, 6].

The sophisticated program of chondrocyte differentiation requires the activation or repression of many genes, which is strictly mediated by transcription factors (TFs), including the SOX (SOX5, SOX6 and SOX9)[7–13], GLI (GLI1, GLI2 and GLI3) [14–17], RUNX (RUNX2 and RUNX3)[18, 19], MEF2C[20], AP1[21] and FOXA[22] family members[23].

SOX9 is the master regulator of chondrocyte differentiation. Chondrocytes cannot form in *Sox9* null mutants and heterozygous mutations in *SOX9* severely disrupt skeletal development, causing campomelic dysplasia [7, 8, 10, 11, 13]. Shortly after mesenchymal condensation, SOX9 cooperates with SOX5 and SOX6 to activate the expression of cartilage matrix genes, e.g., *Col2a1* and *Aggrecan*, positively regulating chondrocyte proliferation, while inhibiting both the progression of these cells to hypertrophy and the osteogenic program [11, 24–26].

The Hedgehog signaling pathway regulates chondrocyte proliferation and hypertrophy through a complex negative feedback loop with the PTHrP signaling pathway [27]. IHH secreted from prehypertrophic chondrocytes (PHCs) activates HH signaling and GLI transcription factors (GLI1/2/3) in proliferating chondrocytes (PCs) via its receptor PTCH1, and stimulates the expression of *Pthrp*. GLI1 functions as an activator which is highly expressed in PCs and perichondrium adjacent to the prehypertrophic and hypertrophic zones. GLI2 has both activator and repressor forms and GLI3 acts as a repressor. GLI2 is expressed in most chondrocytes, but at a lower level in HCs [28]. Binding of PTHrP to its receptor PPR results in activation of PKA and phosphorylation of SOX9 that enhances its transcriptional activity [29], indicating crosstalk between SOX9 and IHH signaling in regulation of chondrocyte proliferation and differentiation. RUNX and FOXA transcription factors are critical regulators of hypertrophic chondrocyte maturation. Mutations in *RUNX2* cause the skeletal disorder, Cleidocranial dysplasia [30]. In mice, inactivation of *Runx2* or *FoxA2*/*FoxA3* causes severe defects in chondrocyte hypertrophy and bone formation [18, 22].

Although these TFs have been studied individually for their importance in chondrocyte differentiation, understanding of how they interact and integrate into a gene regulatory network (GRN) that acts genome wide, is still only emerging and largely limited to addressing control of individual gene expression (reviewed in [23]). For example, *in vitro* transactivation assays in cultured chondrocytes highlight a potential *in vivo* cooperation between GLI1/2 and RUNX2/SMADs in activating *Col10a1* via interaction with its promoter [31]. The Notch signaling pathway transcriptional co-activator, Mastermind-like 1 (MAML1), was reported to enhance the transcriptional activity of RUNX2[32]. Recently genome-wide analyses of SOX9 binding peaks in chondrocytes [33] assisted the discovery that SOX9 and AP1 factors (Jun) co-activate *Col10a1* in prehypertrophic chondrocytes to promote hypertrophy [21]. The SOX proteins are characterized by their dependence on partner factors in controlling cell differentiation [34]. Cooperation of the SOX trio proteins (SOX9, SOX5 and SOX6) controls sequential differentiation of chondrocytes [9, 11, 12, 35–37]. Cooperative interaction between SOX9 and other factors such as AP-1, NFAT, FOXA, RUNX and HOX has also been implicated because their binding motifs are enriched in the SOX9 peaks [21, 33]. It has been reported that GLI1 can regulate *Sox9* via a far upstream enhancer [38] and SOX9 can regulate its own expression via another far upstream enhancer [39]. We previously implicated cooperation between SOX9 and GLI factors in repressing *Col10a1* expression in proliferating chondrocytes [40]. Despite the wealth of information about the individual roles of SOX9 and GLI in regulating chondrocyte differentiation, it is not fully understood about how these TFs together mediate the transition from PCs and PHCs where SOX9 and GLI factors dominate, to HCs controlled by FOXA, RUNX and other factors [40]. Also little is known whether these two factors cooperate to activate chondrocyte genes and if they do, the breadth of potential genes that are cooperatively regulated by these factors.

The need to understand the regulatory mechanisms driving the phases of chondrocyte differentiation in the growth plate has prompted investigations to establish global transcriptomic analyses for gene signatures for the different populations. Prior transcriptomic studies on mouse chondrocyte populations had been narrowly focused on chondrogenic cell lines [41], early stage limb mesenchyme and E13.5 chondrocytes before hypertrophy [42], manually dissected tibia segments [43], or postnatal proliferating and hypertrophic chondrocytes without transition zones [44, 45]. A recent study on the transcriptomes of 217 single cells from the growth plate aimed to reconstruct the spatial and temporal pattern gene expression of individual chondrocytes [46]. However only a limited number of genes were mapped in that dataset and many genes important for chondrocyte differentiation were not detected (e.g. *Ctnnb1*, *Gli2*, *Wnt5a*, *Wnt5b*, etc.). The spatial information on gene expression is also lacking since the cells were not fractionated according to zones.

The limited information on the integration of the GRN that controls the important transitions from one differentiation phase to the next within the growth plate motivated us to develop a comprehensive atlas of gene expression for finely fractionated chondrocyte subpopulations in growth plate. We aimed to use this resource for the analyses and discovery of the complex molecular signatures, differential gene expression patterns, biological processes and pathways operating during the phases of chondrocyte differentiation, especially in the transition into prehypertrophy. We created a searchable library, GP-DGEL (URL: http://www.sbms.hku.hk/kclab/gp.html), that provides sequential and dynamic gene expression information encompassing growth plate chondrocytes at different stages from proliferative to prehypertrophy, early and late hypertrophy. By integrative analysis of the transcriptome and chondrocyte ChIP-seq datasets coupled with functional tests, we find evidence for a dominant role for SOX9-GLI cooperation in proliferating chondrocytes and identify new SOX9 GLI targets. Importantly we find evidence for a model of phase transition of the gene regulatory program from SOX9-GLI cooperation to SOX9-FOXA competition directing chondrocyte differentiation. In this SOX9-GLI-FOXA centric model GRN, SOX9-GLI preferentially activates sets of genes in proliferating chondrocytes. In maturing prehypertrophic chondrocytes the SOX9-GLI nexus begins to fade, so that in full hypertrophy, control is relayed to an alternative set of transcription factors including FOXA2, RUNX2, AP1 and MEF2C.

## Results

### GP-DGEL, a global transcriptome and discovery resource for the growth plate

**Fig 1:**
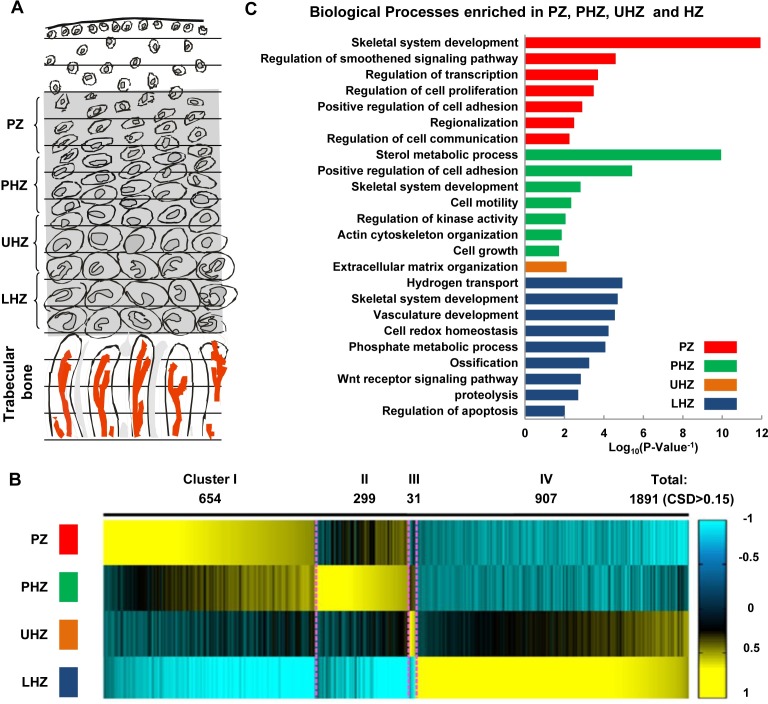
Global gene expression profiling in different chondrocyte populations. (A) 10-day-old mouse growth plates were cryosectioned and mRNA was extracted from the pooled samples of chondrocytes in the PZ, PHZ, UHZ and LHZ. Microarray data were then generated for the expression profiling of 21464 genes in each population of chondrocytes. (B) A total of 1891 genes showed differential expression patterns over the 4 zones with coefficient of standard deviation (CSD) of mRNA levels greater than 0.15. Four major distinct patterns of gene expression over the growth plates were identified in Heatmap by using *K*-Means Clustering. (C) Enriched biological processes representing the main functions of the co-expressed genes in each cluster by GO term analysis.

We generated GP-DGEL, by fractionating the mouse proximal tibial growth plate at postnatal day 10 (P10) into four zones representing chondrocyte sub-populations: PCs in the proliferating zone (PZ), PHCs in the pre-hypertrophic zone (PHZ), early HCs in the upper hypertrophic zone (UHZ) and late differentiated HCs in the lower hypertrophic zone (LHZ) (Fig 1A). Navigating the location and identity of these zones was guided by the morphologies and RT-PCR analyses for the expression of zone-characteristic markers (*Col2a1*, *Col10a1*, *Ppr*, *Ihh* and *Mmp13*; S1A–S1C Fig). Gene expression profiling data from the 4 fractions of biological triplicates were generated for further analysis (S1D Fig).

We defined the set of genes expressed at each stage: a total number of 4799, 4811 and 4879 genes were expressed in PZ, PHZ and HZ (average of the gene expression in UHZ and LHZ) respectively. We categorized all the expressed genes that were commonly or uniquely expressed in PZ, PHZ and HZ (see Methods and S1E Fig). Here, 4792 out of 4886 genes (98%) are commonly expressed showing the “On” state in all three zones. The remainder 94 genes are expressed in only one or two zones (S1 Table). Only one gene, *Fzd9*, was expressed only in PCs. No genes were specifically expressed only in PHCs, as might be expected for cells in transition from proliferation to hypertrophy, but 73 genes were HC-specific.

GP-DGEL allows the detection of variation in gene expression across different zones. Amongst all the expressed genes, 1891 genes (~37%) were differentially expressed with the Coefficients of Standard Deviation (CSD) over the 4 zones greater than 0.15 (S2A Table). The differentially expressed transcription factors included *Sox9*, *Sox5*, *Gli1*, *Gli2*, *Runx3* and *Mef2c* in PZ and PHZ, which is consistent with their known roles in regulating endochondral ossification, affirming the reliability of the dataset. The remaining genes were constantly expressed across the zones, including *Hif1a*, which plays an important role in bone development but is regulated through post-translational modification [47–49].

Using *k*-means clustering analyses to categorize the patterns of the 1891 differentially expressed genes (DEGs), we identified four major clusters: Cluster I genes exhibit decreasing expression from PZ to HZ (654 genes); Clusters II to IV genes are typically most highly expressed in PHZ (299 genes), UHZ (31 genes) and LHZ (907 genes), respectively (Fig 1B; S2A Table). This categorization formed the basis for genome-wide discovery and identification of biological processes, pathways and GRNs that underlie these transition patterns. We tested GP-DGEL for its capacity as a resource for the discovery and functional analyses of signaling pathways, biological processes and transcriptional regulators of chondrocyte differentiation as follows.

### Expression analyses identify differentiation phase-characteristic biological processes

To identify the enriched biological processes and signaling pathways for each cluster, we performed Gene Ontology enrichment analysis. Genes associated with biology process of “skeletal system development” were enriched in PZ, PHZ and HZ, establishing the authenticity of our data (Fig 1C; S3 Table). Genes associated with the processes of “regulation of Smoothened signaling pathway”, “transcription”, and “cell proliferation” were significantly enriched in the PZ (cluster I) and supported a significant role for IHH signaling (mediated by Smoothened and Gli) in proliferating chondrocytes. Genes associated with the processes of “sterol metabolic process”, “cell motility, “actin cytoskeleton regulation” and “cell growth” were most common in the PHZ (cluster II). This agrees with the dramatic changes in chondrocyte size and morphology observed during hypertrophy. Sterol (cholesterol) biosynthesis is required for the processing and maturation of hedgehog ligands and Hedgehog signaling [50]. In explant organ cultures, cholesterol was found to stimulate chondrocyte hypertrophy and bone growth through regulating the expression of *Rora*. Inhibition of cholesterol biosynthesis attenuates chondrocyte enlargement [51] and results in growth retardation with decreased chondrocyte proliferation and *Ihh* expression [52].

The UHZ (cluster III) was enriched for genes associated with the process of “extracellular matrix (ECM) organization”, which is consistent with the transition from synthesis of an ECM rich in collagen II to one where collagen X is the major component [53]. The LHZ (cluster IV) was enriched for “hydrogen transport”, “vascular development”, “cell redox homeostasis”, “phosphate metabolic process”, “ossification” and “regulation of apoptosis”, consistent with late-stage differentiation, cartilage calcification, degradation, vascular invasion, bone formation and chondrocyte to osteoblast trans-differentiation that occur at the chondro-osseous junction[2, 3] where cell cycle re-entry in the process has been implied[4]. The enrichment for “regulation of apoptosis” is intriguing: of the 37 genes highlighted, 16 genes were classed as contributing to "negative regulation of apoptosis" (p-value = 3.5e-18) and 9 genes as contributing to "positive regulation of apoptosis" (p-value = 1.9e-13) such as *Cdkn1a* (p21) a cell cycle regulator and its interacting pro-apoptotic factor Trp53inp1 which may imply complex control that balances apoptosis and survival and control of cell cycle re-entry in the transition from hypertrophic chondrocytes to osteoblasts. Overall the corroboration of genes associated with processes that occur in the relevant zones attests to the quality of the library.

### Constitutive and distinct phase signaling pathways are active in different zones

Many signaling pathways are known to play key roles in coordinating chondrocyte proliferation and differentiation but their relative importance in each phase and sub-population is unclear. A gradient of BMP pathway gene expression has been reported for the rat postnatal growth plate [45] but how this compares with other pathways is not known. To gain global mechanistic insight into the relative scope of signaling action in each zone, we computed the enriched GO terms for the genes involved in canonical signaling pathways: WNT, BMP/TGFβ, FGF, Notch, IGF, Hippo and Hedgehog (S4 Table). We found the expression of the components in WNT, BMP/TGFβ, Hippo, FGF and Notch pathways was not significantly enriched in particular regions over the 4 different zones (Fisher’s exact test p-value > 0.05). In contrast, genes of Hedgehog and IGF pathways were preferentially expressed in PZ and PHZ, suggesting distinctive roles of these signaling pathways in regulating cell cycle progression and the initiation of chondrocyte hypertrophy.

### Genome-wide gene expression analysis identifies skeletal disorder genes

We tested the capacity of GP-DGEL to identify potential associations of the differentially expressed genes with those implicated in mouse skeletal phenotypes and human skeletal diseases in MGI and OMIM databases. A subset of 396 genes, accounting for 20% of the whole list, was associated with abnormal skeletal phenotypes in mouse (S2B Table). 93 genes were associated with human skeletal disorders (S2C Table). To infer the functional significance of the phase-specifically expressed TFs on skeletal development, we ranked the TFs according to the CSD values over 4 zones (S5 Table). Of 76 phasic-specific TFs, 46 (including *Sox9* and *Trps1*) were associated with human skeletal disorders and/or mouse skeletal defects. GP-DGEL can therefore be used to identify new candidate genes of skeletal disorders. An example worthy of further investigation is *Srebf1*, not known to cause skeletal disorders but is implicated as a regulator of cholesterol metabolism and apoptosis (OMIM 184756) [54].

### Motif discovery identifies prominent transcriptional regulators acting in different zones

**Fig 2:**
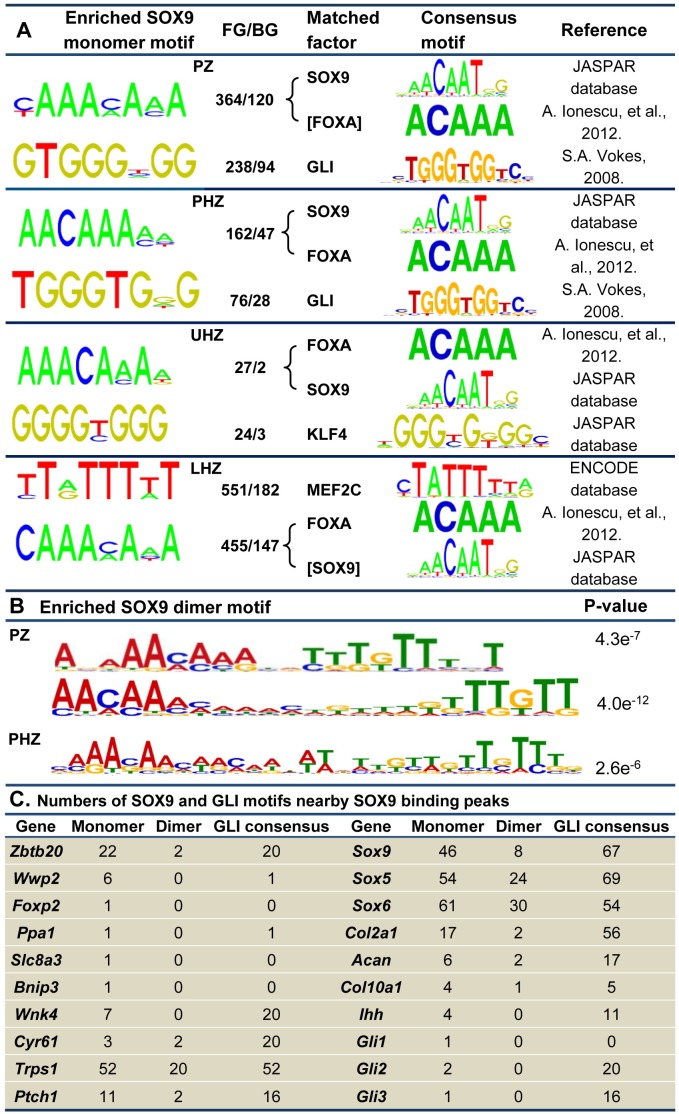
DNA motif enrichment analyses. (A) DNA motifs were identified in the promoter regions of DEGs in the PZ, PHZ, UHZ and LHZ. The output motifs from the DME program were prioritized according to the FG/BG ratio of frequency of occurrence. Of the 50 most highly ranked motifs with at least 2-fold ratio difference, those not expressed or constantly expressed were eliminated from the list. The identified motifs were matched in the TRANSFAC database. Top ranked and matched TFs that were differentially expressed across the growth plate were selected for further analysis. (B) SOX9 motif identified from DME program was utilized for prediction of monomer binding sites within 10kb distance of TSS of DEGs in each zone. The 25bp DNA sequences flanking the monomer SOX9 binding sites were analyzed by using the MEME program. SOX9 dimer motifs were significantly enriched for PZ and PHZ. The width of the spacer sequences in the dimer motifs ranges from 4- to 11-bp for PZ and 10-bp for PHZ genes, counted from the last base pair position of the 5’-AACAA-3’ SOX9 binding core consensus. (C) The numbers of SOX9 monomer motifs (Regular TRANSFAC consensus, COL2C1, COL2C2 and COL2C3), the SOX9 dimer motifs and the GLI binding motifs that are located within 250-bp from the SOX9 binding peaks identified in DEGs.

The large sets of genes sharing phasic-specific expression patterns imply the presence of a coordinated transcriptional program at each phase. For an unbiased identification of phase specific transcriptional regulators, we performed de novo motif enrichment analysis in the promoter regions for the genes in each cluster using the computer program Discriminating Motif Enumerator (DME), MotifClass and MatCompare [55–58]. The most enriched TF binding motifs include SOX9 and GLI (GLI1, GLI2 and GLI3) in the PZ and PHZ; SOX9/FOXA and KLF4 in the UHZ; and MEF2C and FOXA motifs in the LHZ (Fig 2A). The core consensus binding motifs for FOXA factors (FOXA1, FOXA2 and FOXA3) and SOX9 comprise highly similar AT-rich sequences (ACAAA-like for FOXA; ATTGT-like for SOX), raising the possibility that these factors compete for binding in regulating gene expression [22].

The over-representation of a GLI motif in the PZ and PHZ genes agrees with the known action of Hedgehog signaling in PCs. *Gli1*, itself a target of Hedgehog signaling, is most highly expressed in the PZ, while the cytoplasmic GLI3 repressor may transform into an activator in the presence of IHH [59]. KLF4 motif enrichment in the UHZ cluster could imply a role in promoting hypertrophy which would be consistent with its capacity to reprogramme dermal fibroblasts in concert with SOX9 and cMYC [60]. In the LHZ cluster, we detected enrichment for the binding motif for MEF2C, a vital regulator of chondrocyte hypertrophy that is required for the proper expression of *Col10a1*, *Runx2* and *Vegf* [20]. Beyond the chondrocyte fate, KLF4 and MEF2C may prime the lineage progression of hypertrophic chondrocytes to osteoblasts [3, 61, 62].

SOX9 functions as a dimer in chondrocyte differentiation [63]. To predict the degree to which SOX9 dimer/monomer binding motifs were utilized in chondrocyte gene regulation, we screened for evolutionarily conserved SOX9 binding sites located within 10kb from the transcriptional starting site (TSS). Using the MEME program [64] for long consensus motif analysis, we identified SOX9 dimer motifs with varied length of spacer sequences (Fig 2B). The most enriched SOX9 dimer motifs were identified for PZ and PHZ genes (p-value<1.0e-5). In proliferating chondrocytes, SOX9 dimer motifs were associated with the genes which it activates (e.g. *Col2a1*) or represses (e.g. *Col10a1*) [24, 40, 65]. The length of the spacer sequences in the dimer motifs ranges from 4 to 13-bp in the PZ and 4 to 16-bp in the PHZ genes (S6 Table), raising the question whether the variation in the linking sequences could confer different specificity of co-binding of partner factors with SOX9 dimers [66, 67]. For LHZ where SOX9 protein level dropped to undetectable level, no significant SOX9 dimer binding motifs were identified.

### Identification of SOX9 candidate target genes in the growth plate

The enrichment of SOX9 binding motifs in the DEGs (Fig 2A and 2B) is consistent with the vital roles of SOX9 in regulating phasic gene expression and helps identification of target genes regulated by SOX9 at each stage of chondrocyte differentiation. We searched the evolutionarily conserved noncoding DNA elements across 30 vertebrates in gene promoter, intergenic, intronic and 3’- UTR regions for putative SOX9 monomer and dimer binding sites (S7A–S7D Table). To identify functional binding sites, we integrated the bioinformatics predictions with the SOX9 ChIP-seq dataset from mouse neonatal rib chondrocytes [33]. Overall, 503 genes out of 654 in the PZ cluster, 250 out of 299 in the PHZ cluster, 24 out of 31 in the UHZ cluster and 664 out of 907 in the LHZ cluster were found to harbor at least one SOX9 binding region (SBR) (S8A1–S8B2 Table), consistent with the major role of SOX9 in regulating chondrocyte differentiation. SOX9 can act as both an activator and a repressor in PCs [40]. Therefore the genes identified by this analysis could be either activated or repressed by SOX9.

**Fig 3:**
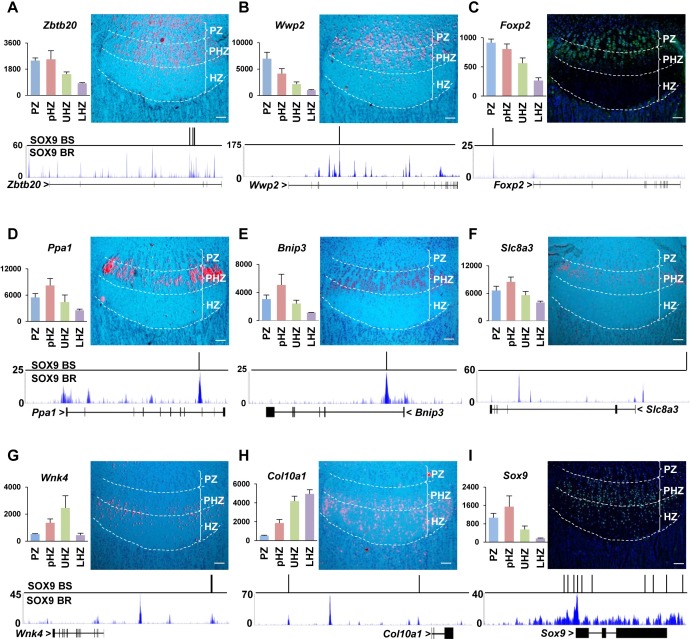
Expression patterns of putative SOX9 target genes and SOX9 binding regions in their genomic loci. (A-I) Genes with SOX9 binding motifs located within 200bp from SOX9 ChIP-seq peaks (Generated from newborn rib chondrocytes) were selected for validation. *In vivo* expression patterns of potential SOX9 targets were validated by in situ hybridization: *Zbtb20* (A), *Wwp2* (B), *Ppa1* (D), *Bnip3* (E), *Slc8a3* (F), *Wnk4* (G) and *Col10a1* (H); or immunostaining: FOXP2 (C) and SOX9 (I), revealing similar expression trends with the microarray data as shown on the left side. The PZ, PHZ and HZ were separated by the white-dot lines. (Bar = 100μm). Predicted SOX9 binding sites (BS: binding site) and SOX9 ChIP-seq signals (BR: binding region) (Green) are shown under each gene.

We selected those genes with predicted SOX9 binding sites located within 250-bp from the SOX9 ChIP peaks as potential SOX9 targets. Multiple copies of monomer and dimer sites near the SOX9 binding peaks were identified for known SOX9 targets *Sox9*, *Sox5*, *Sox6*, *Col2a1*, *Acan* and *Col10a1* (Fig 2C; S7E and S7F Table). In the *Col10a1* locus, we found a SOX9 binding peak 4.4 kb upstream of the TSS (Fig 3H), where an element has been shown to mediate repression by SOX9 in non-hypertrophic chondrocytes [22, 40]. These data affirm the validity of our approach.

We identified several potential SOX9 targets (*Zbtb20*, *Wwp2*, *Foxp2*, *Ppa1*, *Slc8a3*, *Bnip3* and *Wnk4*). By *in situ* hybridization or antibody staining on proximal tibia growth plate, we confirmed the expression patterns of these targets (Fig 3A–3I) as corresponding with the regions identified in GP-DGEL. These potential SOX9 targets may function in different steps of chondrocyte differentiation and endochondral bone formation. For instance, *Wwp2* (Fig 3B) has been identified as a direct SOX9 target during palatogenesis [68]. Interestingly many genes encoding major components of the IHH signaling pathway were identified as potential SOX9 targets in our study, including *Ihh*, *Ptch1*, *Gli1*, *Gli2* and *Gli3* (Fig 2C), which is consistent with the enrichment for GLI binding motifs for the PZ and PHZ clusters (Fig 2A).

### Functional validation of new SOX9 targets

**Fig 4:**
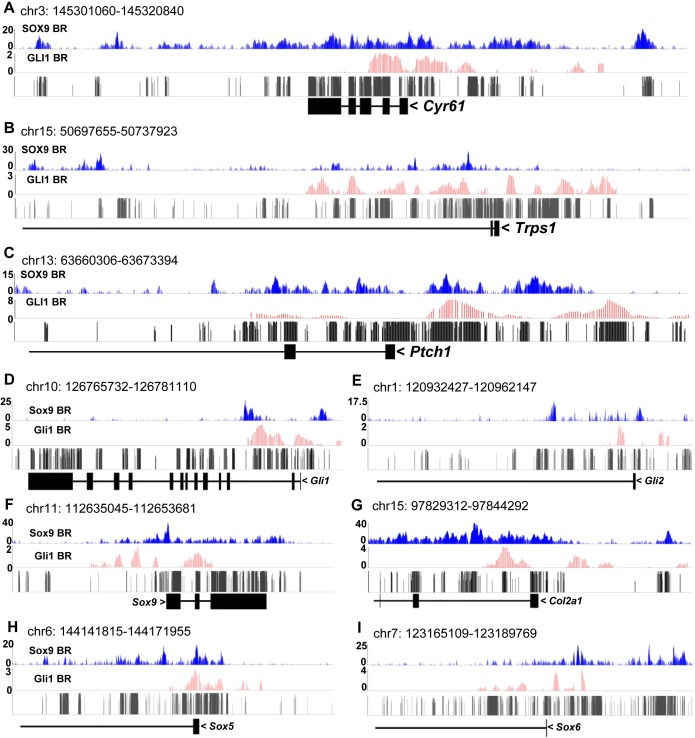
Predicted regulation by SOX9 and GLI1 cooperation. (A-C) SOX9, GLI1 (Generated from E11.5 developing limb) ChIP-seq signals and conservation score in the loci of *Cyr61*, *Trps1* and *Ptch1*. BS: binding site; BR: binding region. (D-I) SOX9, GLI1 ChIP-seq signals and conservation score in the loci of Hedgehog target genes (*Gli1* and *Gli2*) and Sox9 target genes (*Sox9*, *Col2a1*, *Sox5* and *Sox6*).

To validate SOX9 binding under the SOX9 peaks, we performed *in vivo* ChIP-qPCR assays on three candidates: *Cyr61*[69, 70], *Trps1* [59, 71] and *Ptch1* (S9 Table), with an *Aggrecan* enhancer situated 10kb up-stream of TSS as a positive control [12]. The ChIP-qPCR results showed that SOX9 binds to the promoters of *Cyr61* and *Ptch1*, and the intron 1 of *Trps1* (S2A–S2D Fig). Within the regions covering the validated binding sites, we detected SOX9 binding peaks (Fig 4A–4C), indicating that these genes are direct SOX9 targets.

**Fig 5:**
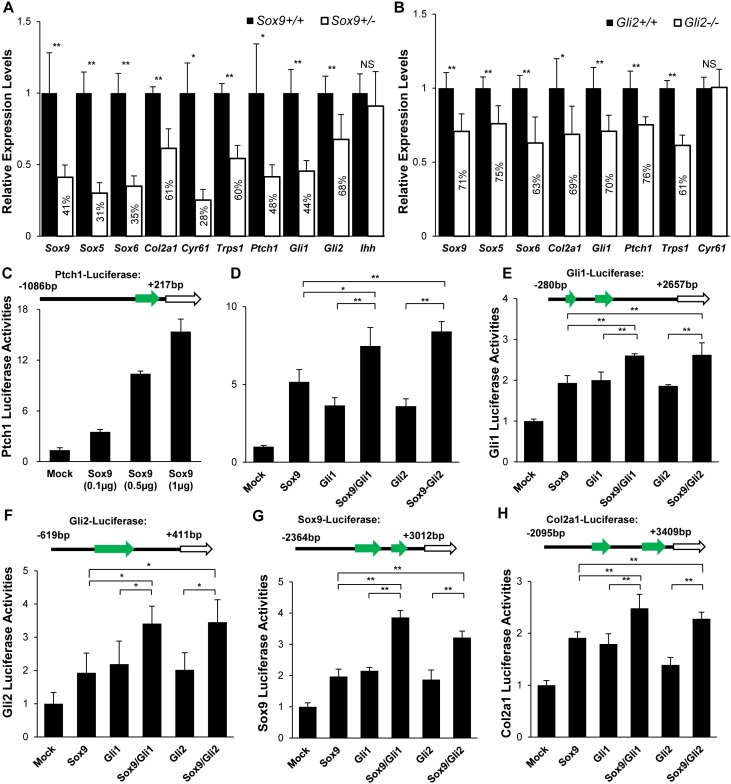
Functional validation of SOX9 and GLI cooperation. (A) Relative expression levels of SOX9-GLI common target genes in *Sox9*^*+/+*^ and *Sox9*^*+/-*^ limb chondrocytes (E13.5). (B) Relative expression levels of SOX9-GLI common target genes in *Gli2*^*+/+*^ and *Gli2*^*-/-*^ limb chondrocytes (E13.5). *Gapdh* was used as the endogenous control. The error bar represents the standard deviation of the relative expression level of each gene in independent triplicate. (C) SOX9 activated the expression of *Ptch1* promoter in a dosage dependent manner in transiently transfected ATDC5 cells. (D-H) SOX9 increased GLI-dependent activation of *Ptch1*, *Gli1*, *Gli2*, *Sox9* and *Col2a1* regulatory elements in transiently transfected ATDC5 cells. Expression plasmids for *Sox9*, *Gli1*, *Gli2* and their combination were cotransfected as indicated below the bars. Equal amounts of expression plasmids were used for each well. **: p-value < 0.01; *: p-value < 0.05; NS: Non-significant.

To test whether the expression of these candidates is associated with SOX9 activity, we compared their expression levels in wild type (*Sox9*^*+/+*^) and heterozygous null (*Sox9*^*+/-*^) mutant littermates in embryonic day 13.5 (E13.5) limbs, when the limb abnormality is minimal [72]. Expression of known SOX9 targets (*Sox9*, *Sox5*, *Sox6* and *Col2a1*) was down regulated in *Sox9*^*+/-*^ mutants compared with wild type littermates (Fig 5A), consistent with the dosage requirement for SOX9. Expression of *Cyr61*, *Trps1*, *Ptch1*, *Gli1* and *Gli2* was reduced by approximately 50% in *Sox9*^*+/-*^ mutants (Fig 5A), and *Gli3* expression has been reported to be reduced in *Sox9*^*+/-*^ mutants [35], indicating that SOX9 positively regulates these genes. *Ihh* is expressed in PHCs [14], and its expression is not significantly changed in *Sox9*^*+/-*^ mice, consistent with the previous finding that the expression of *Ihh* is not affected by *Sox9* heterozygous mutation [72].

Since *Ptch1* is down-regulated in *Sox9*^*+/-*^mutants, we tested whether *Ptch1* is transcriptionally regulated by SOX9. Using the chondrogenic cell line ATDC5, we found SOX9 transactivated a luciferase reporter driven by regulatory sequences in the *Ptch1* promoter region containing a SOX9 binding peak in a dosage-dependent manner in (Fig 5C), indicating that SOX9 directly regulates the expression of *Ptch1*.

### Predicted cooperative transactivation by SOX9 and GLI

The enrichment for SOX9 and GLI motifs in the PZ and PHZ clusters (Fig 2A), the co-expression of *Sox9* and *Gli1* in the PCs (S3A and S3B Fig), the known GLI activation of *Ptch1* [73] and the cooperative repression of *Col10a1* by SOX9-GLI3^R^ [40] raise the possibility of a substantial role for cooperation of SOX9 with GLI in activating gene expression. To investigate whether SOX9 and its targets are co-regulated by GLI factors, we screened the phasic DEGs for putative GLI binding sites (S7E Table). Abundant GLI consensus motifs were found near SOX9 peaks in *Sox9* itself and SOX9 target, in particular *Sox9*, *Sox5*, *Sox6* and *Col2a1* (Fig 2C). Since it has been reported that cells derived from a common progenitor lineage share similar genome-wide epigenetics and TF binding profiles[74], we integrated the bioinformatics predictions with the SOX9 ChIP-seq from newborn rib chondrocytes (S8A1 and S8A2 Table), GLI1 (S8C1 and S8C2 Table) and GLI3 (S8E1 and S8E2 Table) ChIP-chip datasets from E11.5 developing limbs[73] to check whether in principle, these TFs could bind to the putative common target genes.

Binding regions for SOX9 (SBR), GLI1 and GLI3 (GBR) were found in 1426, 699 and 1421 phasic DEGs respectively (SOX9, S8B1 and S8B2 Table; GLI1, S8D1 and S8D2 Table; and GLI3, S8F1 and S8F2 Table). Among these, 721 of 1426 SOX9-targeted DEGs (51%) harbored at least one SOX9/GLI linked binding region (SGBR) with an inter-peak distance shorter than 250-bp (S10A–S10D Table). The genes that were most enriched for SGBRs include the known SOX9 targets *Sox9*, *Sox5*, *Sox6* and *Col2a1*. Interestingly substantial over-representation of putative SOX9/GLI common targets was found for the PZ, PHZ and UHZ clusters compared to genes in the LHZ cluster (p-value<0.01), consistent with the expression pattern of SOX9 protein which spans the PZ, PHZ and persists into the UHZ [26]. The correlation of SGBRs with phasic gene expression decreased as the inter-peak distance increased (S10A1, S10D2 and S10D2 Table). Correlation was also found between phasic-specific genes and SGBRs that were located in the intergenic regions (p-value = 0.0032, S10A3 Table), suggesting SOX9-GLI may also mediate long-range regulation.

### SOX9 and GLI cooperatively transactivate gene expression

These bioinformatics predictions suggest that SOX9 and GLI factors cooperate to regulate common targets in PCs and PHCs. To test these predictions we examined the SOX9 ChIP-seq data and found that the SOX9-bound regions in *Trps1* and *Ptch1* loci were co-localized with the GLI1 binding peaks (Fig 4B and 4C). We tested the ability of SOX9 and GLI singly and in combination to transactivate expression of the *Ptch1-*luciferase reporter vector. Both SOX9 and GLI1 could drive the expression of the *Ptch1*-luciferase reporter (Fig 5C and S3C–S3F Fig). We also detected SOX9 binding peaks in the *Gli1* and *Gli2* loci (Fig 4D and 4E). In *Sox9*^*+/-*^ mutants, the expression of *Gli1* and *Gli2* was down-regulated (Fig 5A), indicating that these genes may be regulated by SOX9.

Firstly we tested for the cooperative control of GLI on SOX9 targets. We found GLI1 peaks in SOX9 target genes, including *Sox9*, *Col2a1*, *Sox5* and *Sox6*, which are close to SOX9 binding regions (Fig 4F–4I). To test whether the expression of SOX9 targets is affected by removal of the GLI activator, we compared their expression levels in *Gli2*^*+/+*^ and *Gli2*^*-/-*^ littermates. In *Gli2*-null mutants, with the exception of *Cyr61*, the SOX9 targets *Sox9*, *Col2a1*, *Sox5*, *Sox6* and *Gli1*, *Trps1* and *Ptch1* were markedly downregulated (Fig 5B), consistent with cooperative regulation by SOX9 and GLI.

We next tested the cooperative activity of SOX9 and GLI in regulating *Sox9*, *Col2a1*, *Ptch1*, *Gli1* and *Gli2* by transactivation assays using luciferase reporters driven by genomic fragments containing at least one SGBR. GLI1 and GLI2 transactivated the *Ptch1*, *Gli1* and *Gli2* reporters. This transactivation activity was significantly enhanced by SOX9 (Fig 5D–5F). SOX9-dependent transcriptional activation of *Sox9* and *Col2a1* reporters was enhanced by GLI1 and GLI2 (Fig 5G and 5H), confirming the addictive action of SOX9 and GLI activators in the regulation of common target genes.

### Transition from SOX9-GLI cooperation in PCs to SOX9-FOXA competition in HCs

**Fig 6:**
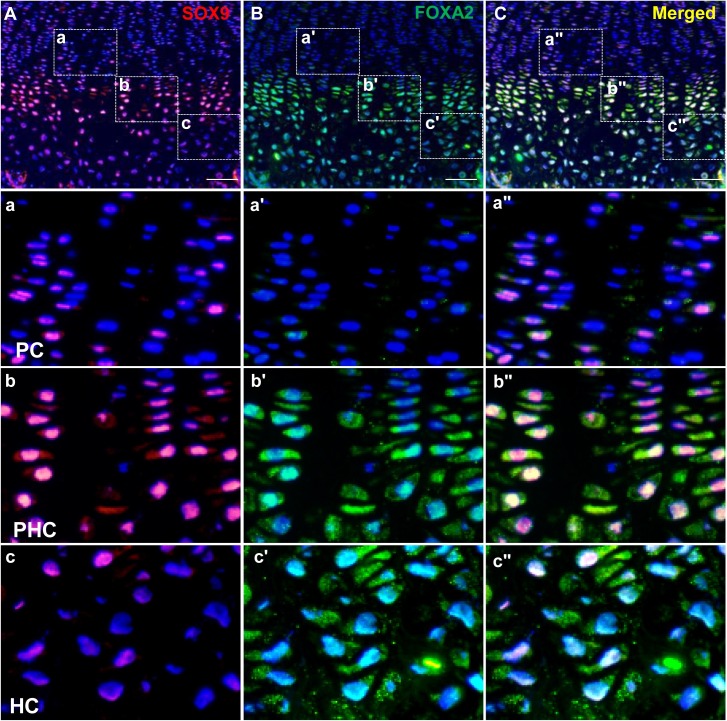
Co-localization of SOX9 and FOXA2 in the growth plate. (A-C) *In vivo* expression patterns of SOX9 (A, red), FOXA2 (B, green) and co-localization (C) were shown on the cryosectioned growth plate (P10). Boxed regions of PCs (a-a"), PHCs (b-b") and HCs (c-c") were shown in higher magnification to demonstrate the differential expression and co-localization of SOX9 and FOXA2. (Bar = 100μm).

While SOX9 and GLI factors play key roles in the GRN of PCs and PHCs, they are not expressed in hypertrophic chondrocytes in the LHZ (Fig 6A, S3A and S3B Fig). We therefore sought to gain insight into the GRN that mediates the transition from proliferation to prehypertrophy and hypertrophy. It is notable that *Foxa2*, a critical regulator of hypertrophy [22], is mainly expressed in PHCs and HCs (Fig 6B). Interestingly, SOX9 and FOXA2 are co-expressed in PHCs and early HCs (Fig 6C). We analyzed the published FOXA2 ChIP-seq dataset [75] which has been used in other studies to identify multiple target genes in the notochord [76], and found multiple FOXA2 binding peaks in the *Sox9* promoter and distal gene regulatory elements (S8G Table). As *Sox9* expression diminishes in the UHZ whilst *Foxa2* is robustly expressed, it is possible that FOXA2 represses *Sox9* expression.

**Fig 7:**
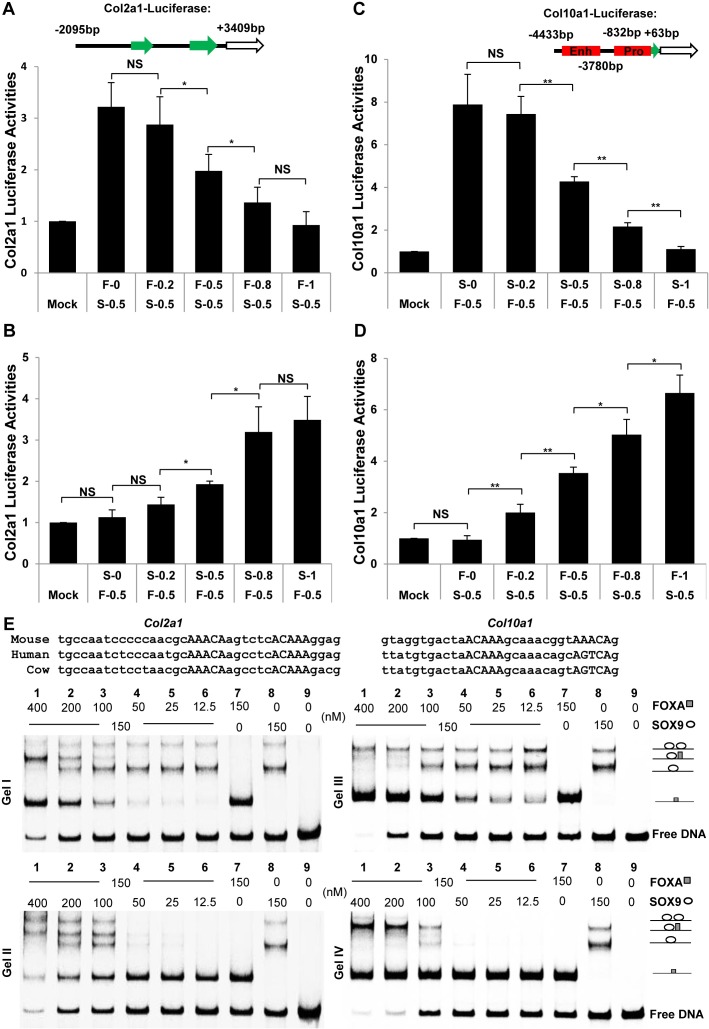
Competition between SOX9 and FOXA2 during chondrocyte differentiation. (A-D) Competition between SOX9 and FOXA2 on *Col2a1* intron 1 and *Col10a1* enhancer by luciferase assay in ATDC5. Enh: enhancer; Pro: promoter. The amount of SOX9 was kept constant and FOXA2 was increased and vice versa. (E) EMSA was performed using probes for the *Col2a1* intron I (Gel I and II) and *Col10a1* enhancer (Gel III and IV), and SOX9 and FOXA proteins at indicated concentrations. The sequences from *Col2a1* intron I and *Col10a1* enhancer used in the EMSAs are shown in the upper panel, and the SOX9/FOXA binding motifs are indicated by capital letters. **: p-value < 0.01; *: p-value < 0.05; NS: Non-significant.

As FOXA2 and SOX are co-localized in PHCs and early HCs and bind closely related AT-rich DNA elements [22], FOXA2 and SOX9 could compete for binding sites and alter the dynamics of the regulatory phase. To study the relationship between these two factors, we expressed SOX9 and FOXA2 in ATDC5 cells and examined their impact on the transactivation of two established SOX9 targets, *Col2a1* and *Col10a1*, using promoter/enhancer-driven luciferase reporter expression. As expected, SOX9 transactivated the expression of a *Col2a1*-luciferase reporter (Fig 7A). However, this transactivation was progressively weaker with increasing amounts of FOXA2. Notably, FOXA2 alone did not transactivate the *Col2a1* reporter. The reporter was progressively activated with increasing amounts of SOX9 (Fig 7B).

SOX9 represses the expression of *Col10a1* by direct binding to the conserved regulatory region located between −4.3 and −3.6 kb of the mouse *Col10a1* gene [40]. We tested the ability of FOXA2 to activate the expression of luciferase driven by the *Col10a1* promoter (-832bp to +68bp) with the enhancer region (-4433bp to -3780bp). FOXA2 alone could transactivate the *Col10a1-*luciferase reporter and this activation was gradually dampened with increasing amounts of co-transfected SOX9 (Fig 7C). SOX9 alone did not transactivate the *Col10a1* reporter and the repression was released with increasing amounts of FOXA2 (Fig 7D).

To test further the transcriptional competition between SOX9 and FOXA, we selected known regulatory regions from the *Col2a1* and *Col10a1* loci [24, 40, 77] containing SOX9/FOXA binding motifs and carried out electrophoretic mobility shift assay (EMSA) with homogenously purified FOXA and SOX9 protein constructs (Fig 7E, Gel I-IV). We found that both FOXA and SOX9 effectively associated with the *Col2a1* and *Col10a1* sequences with FOXA migrating as a monomer (Fig 7E, Gel I-IV, lane 7) whereas SOX9 migrated as a monomer or dimer under equilibrium conditions (Fig 7E, Gel I-IV, lane 8). The SOX9 monomer fraction predominates at the tested concentration suggesting that the homodimer cooperativity is profoundly weaker than for canonical SOXE DNA elements in the reverse-forward (ACAATGN_3-5_CATTGT) configuration [66]. On the *Col2a1* element, SOX9/FOXA heterodimer fractions appeared under conditions when the FOXA monomer is also formed suggesting that FOXA is able to interact with DNA bound by SOX9 monomers (Fig 7E, Gel I, lane 2 and 3). At high FOXA concentration when DNA probes become limiting, the SOX9 monomer disappear and the FOXA monomer and SOX9/FOXA heterodimer become dominant (Fig 7E, Gel I, lane 1). The dimeric SOX9/DNA complex persisted even at very high FOXA concentrations suggesting a highly stable association of dimeric SOX9. In the inverse experiment when the FOXA concentration is fixed and SOX9 is increased, the SOX9/FOXA heterodimer is formed equally well and at high Sox9 concentration the Sox9 homodimer is formed at the expense of the FOXA monomer (Fig 7E, Gel II), suggesting that SOX9 and FOXA can from heterodimers on *Col2a1* DNA in an un-cooperative fashion but SOX9 homodimers and FOXA monomers are incompatible and compete. On the *Col10a1* element, SOX9 also forms a homodimer with similar efficiency as on *Col2a1* DNA whilst FOXA binds monomerically. However, a SOX9/FOXA heterodimer is barely visible on this element (Fig 7E, Gel III and IV, lane 1–3). Interestingly, the presence of FOXA counteracts the formation of monomerically bound SOX9/DNA complexes but favors the formation of dimeric SOX9/DNA complexes (Fig 7E, Gel III and IV, compare lanes 1–3 with lanes 8). This indicates that dimeric SOX9 more effectively resists competition by FOXA than monomeric SOX9. Together, these findings demonstrate that FOXA and SOX9 possess the capacity to associate with highly similar DNA sequences and indicate that competition between SOX9 and FOXA is a plausible mechanism for the transcriptional switches during chondrogenesis.

## Discussion

### GP-DGEL and an integrated approach for gene discovery

In our study we have aimed to provide insights into the gene expression dynamics and gene regulatory network that guide chondrocytes through their phases of differentiation in the growth plate. Although the cells in each region were not pure populations, especially in the LHZ which is adjacent to the primary ossification center with vascular invasion, the expression profiles of many chondrogenic markers (*Sox9*, *Sox5*, *Sox6*, *Wwp2*, *Col2a1*, *Col9a1*, *Acan*, *Comp*, *Ihh*, *Ptch1*, *Gli1*, *Gli2*, *Ppr*, *Fgfr3*, *Igf1*, *Bmp6*, *Wnt5b*, *Dkk1*, *Cdkn1c*, *Mef2c*, *Bmp2*, *Col10a1*, *Mmp9*, *Mmp13*, *et al*) did show high consistency with the published data. Cognizant of the potential limitations we have validated the expression of the novel genes (*Zbtb20*, *Foxp2*, *Slc8a3*, *Ppa1* and *Bnip3*). Therefore analysis of these microarray data still provides vast transcriptomic information on chondrocyte differentiation. Towards that end, we developed a library of differentially expressed genes, GP-DGEL that has fine spatial resolution and global transcriptomic coverage, allowing systematic analyses of the genes that regulate transition between these phases. GP-DGEL is a valuable resource to complement efforts to identify causative mutations in skeletal dysplasia and predict the underlying GRN. This is illustrated by our correlative analyses of the 1891 DEGs with the MGI and OMIM databases, which identified genes associated with mouse and human skeletal disorders and additional candidates (S2 Table). GP-DGEL has also enabled the identification of new gene signatures. Many of the DEGs remain poorly studied in chondrocytes. Integration of the dataset with global ChIP-seq data allows the identification of target genes for TFs, singly and in combination, thereby revealing cooperative activities. Using this approach we identified new targets for SOX9 and evidence for SOX9-GLI cooperation.

We validated several of the predicted SOX9 targets (*Cyr61*, *Trps1*, *Ptch1*, *Gli1 and Gli2)* by functional assays. The downregulation of *Cyr61*, *Trps1 and Gli2* in *Sox9*^*+/-*^ chondrocytes in a recent report [35] is in agreement with our data. The presence of SOX9 peaks associated with these genes in SOX9 ChIP-seq data from rat chondrosarcoma cells is also consistent with direct regulation [37]. We also confirmed the expression patterns of other potential SOX9 targets (*Zbtb20*, *Wwp2*, *Foxp2*, *Ppa1*, *Bnip3*, *Slc8a3 and Wnk4*) that were identified based on the presence of associated SOX9 binding peaks (Fig 3). These genes are candidates for functional studies. An example is *Wnk4*, which is expressed in late PHCs and early HCs (Fig 3G). WNK4 is the major regulator of the Na-Cl co-transporter in the kidney, a regulator of adipogenesis and energy metabolism and a causal gene for pseudohypoaldosteronism type II [78–80], but has no known role in chondrocyte hypertrophy.

### Cooperative regulation of chondrocyte differentiation phases by SOX9 and GLI

A major outcome of the integrated approach is the identification of genes that are co-regulated by both SOX9 and GLI factors. *Zbtb20*, highly expressed in PHCs and downregulated in the UHZ, is a potential SOX9-GLI target since SOX9 and GLI binding peaks were identified in the locus (Fig 3A). Ablation of *Zbtb20* in chondrocytes results in an expanded HZ, and delayed vascularization [81], consistent with a role downstream of SOX9 in regulation of the transition from prehypertrophy to hypertrophy [26]. This function may additionally require co-regulation of SOX9-GLI. Other predicted targets that can be followed up in functional analyses include *Fgfr3*, *Igf1r*, *Bmp6*, *Wnt5a* and *Ror2*, which are the direct targets of GLI1 and/or GLI3, among which *Fgfr3*, *Igf1r* and *Bmp6* are also targeted by SOX9 (S8 Table), indicating SOX9-GLI interacts with FGF, IGF, BMP and WNT signaling for regulating chondrocyte proliferation and differentiation. Although in the luciferase assays, SOX9 show higher transactivation potential on *Ptch1* reporter comparing to GLI1 and GLI2 (Fig 5D), their transcriptional activities are comparable on other Hedgehog targets including *Gli1* and *Gli2* (Fig 5E and 5F). Since the regions used in these experiments only comprise a short representative fragment containing SOX9 and GLI binding regions, the level of transactivation achieved should not be taken as an absolute quantification of the degree of activation of the gene *in vivo*. Therefore we cannot conclude that SOX9 is a more potent factor for *Ptch1* then GLI1 and GLI2. But we can confirm that *Ptch1* is a common target of SOX9 and GLI.

### Transcriptional network model regulating chondrocyte differentiation phases

**Fig 8:**
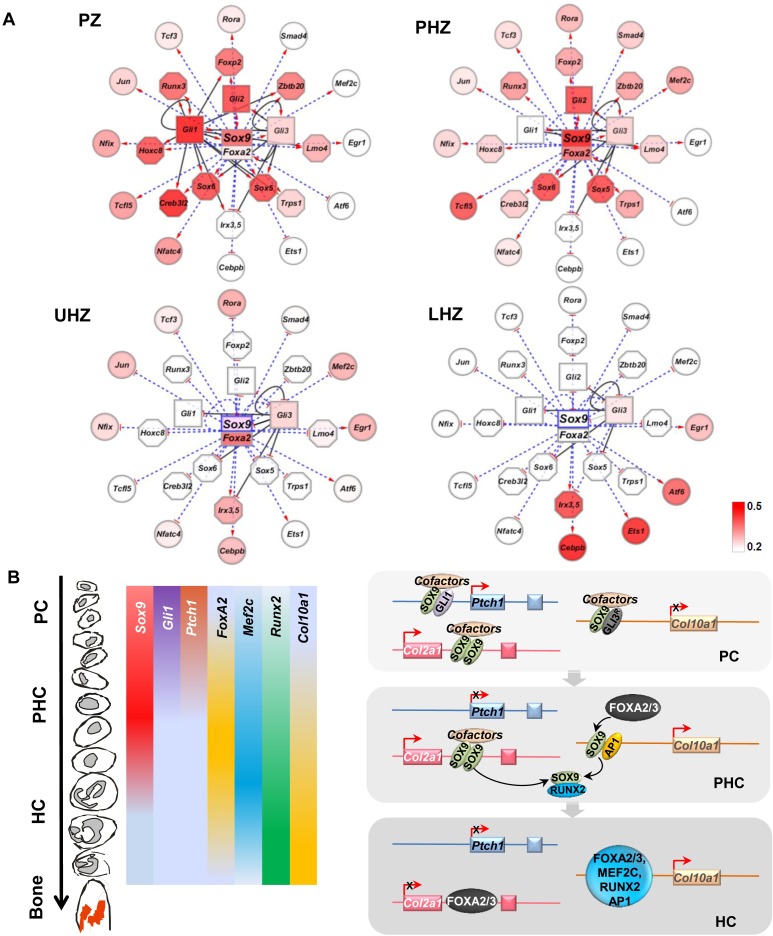
Model of the growth plate GRN directing chondrocyte differentiation. (A) Stage-specific gene transcriptional programs regulating chondrocyte proliferation (PZ), pre-hypertrophy (PHZ), early hypertrophy (UHZ) and terminal differentiation (LHZ). Each node represents a transcription factor gene or its protein product. All the genes in the network are SOX9 targets identified from the ChIP-sequencing experiment. Each solid line represents an identified GLI-target gene relationship, pointing from GLI1/GLI3 to the target. The TF genes that were targeted both by SOX9 and GLI1/GLI3 are in the inner circle of the network, while those targeted only by SOX9 were placed in the outer circle. The dotted blue line indicates the presence of the predicted evolutionarily conserved SOX9/FOXA2 binding consensus in the target genes. The changes of color codes in the gene nodes from dark to light red and white indicate the dynamics of gene expression levels during the chondrocyte state transition (red representing high expression level; white, low expression). (B) Schematic model of a SOX9-GLI-FOXA phasic GRN coordinating chondrocyte differentiation and transitions in the growth plate.

Our analyses of the transcriptome and functional assays have implicated SOX9, GLI and FOXA as key regulators mediating differentiation transitions in the growth plate. Building on this finding, we went further to infer the wider interaction network mediated by these factors. By integrating data in GP-DGEL with SOX9, GLI1 and GLI3 ChIP-seq datasets, we found evidence for a regulatory network centered on SOX9-GLI-FOXA (Fig 8A). The GRN presents the progressive changes in expression of TFs as chondrocyte transition from proliferating to late differentiated states. Since SOX9 and GLI are highly expressed and their DNA binding regions are most enriched in the cluster of PZ genes (Fig 2A), we placed SOX and GLI families in the center of the network (Fig 8A). The common targets of SOX9 and GLI harboring at least one SGBR were placed in the inner circle of the network, while the genes that were targeted singly by SOX9 or GLI were arranged in the outer rim. Multiple binding regions for SOX9 and GLI factors were identified in the promoters and the intergenic regions of *Gli1* and *Gli2* and also the SOX trio of *Sox9*, *Sox5* and *Sox6* (S8 Table). The model therefore predicts the existence of regulatory feedback loops in which *Gli1* and *Gli2* are the targets of SOX9 and vice versa.

We next focused on the key transition from PHZ to UHZ and LHZ. The expression of SOX9 single target genes without GLI binding peaks in this outer rim, including transcription factors *Mef2c*, *Rora*, and *Tcf3*, showed little change in the transition from PHZ to UHZ. Exceptions included *Irx3*, *Irx5*, *Cebpb*, *Ets-1*, *Atf6*, and *Egr1*, which were not expressed in the PZ and PHZ but progressively increased in the UHZ and LHZ as SOX9 expression was down-regulated in the LHZ. These genes may be negatively regulated by SOX9 since SOX9 is both an activator and a repressor. SOX9 represses *Col10a1* expression in PCs[40] and osteoblastic gene expression in HCs[26]. The coincident increasing gradient of expression of *Foxa2* in the PHZ and UHZ, and the shared binding consensus of SOX9 and FOXA2, are consistent with the role of FOXA2 in activating the hypertrophic program[22]. Whether the same motif mediates transactivation and repressor activities is unclear. The SOX9 motif was also identified in the LHZ where SOX9 protein is absent, suggesting this motif may mediate the activity of the FOXA factors (FOXA2 and FOXA3), which are expressed in HCs and may compete with SOX9 for the SOX/FOXA motif[22].

### A SOX9-GLI-FOXA GRN controlling phases of chondrocyte differentiation

SOX9 is known to auto-regulate itself through several long-range enhancers [82] and GLI factors can activate Sox9 through direct binding to a far upstream enhancer[38]. From ChIP-chip data, GLI1 input was found in the *Sox9* promoter region (Fig 4F), suggesting that proximal chromatin interaction by GLI activator may also contribute to *Sox9* expression. Our data indicate that in the context of growth plate cartilage, IHH signaling targets *Sox9* as well as its transcriptional targets through GLI factors, and vice versa, to promote stage-specific chondrocyte differentiation, consistent with previous studies which found that the expression of *Sox9* was induced by HH signaling articular cartilage chondrocytes[83] and retinal explants[84].

The cooperative action of SOX9 and GLI factors may reflect a wider application of cooperation between SOX and GLI factors in other systems. It is interesting that in neural tube patterning, cell fate determination requires both SOX2 and GLI1 inputs [85]. In pancreatic ductal adenocarcinoma, both SOX9 and GLI1 are important to maintain the malignant phenotype of cancer stem cells. Suppression of either SOX9 or GLI1 by siRNA reduces the expression of *Sox9*, *Gli1* and *Gli2* [86]. In primary chondrocytes, SOX9 up-regulates the expression of *PthrP* through direct binding to its promoter region via collaboration with GLI2 [87]. Even when Hedgehog signaling was blocked by cyclopamine, overexpression of *Sox9* could still increase the expression of *Ptch1*, which is consistent with our finding that SOX9 positively regulates the transcription of *Ptch1* without affecting *Ihh* expression. This report also demonstrated that SOX9 can directly bind to GLI2 *in vitro*[87], thus their direct interaction for cooperation *in vivo* warrants further investigation.

We propose a SOX9-GLI-centric model in which SOX9 and IHH signaling initiate control of chondrocyte differentiation phases, especially in the PZ and PHZ (Fig 8B). Upon transition to PHC, down-regulation of GLI repressor (due to Hedgehog signaling) allows higher levels of activator SOX9 together with RUNX2, and increased expression of MEF2C and FOXA2/3 which promote hypertrophic differentiation exemplified by *Col10a1* expression[26]. We demonstrated that FOXA proteins compete with SOX9 for the binding to regulatory elements derived from *Col2a1* and *Col10a1*. These data imply FOXA2 competition with SOX9 is important for the switch to the down-regulation of *Col2a1* and activation of *Col10a1* in HCs. We propose that in the presence of high SOX9 level, particularly in its highly stable homodimeric form, FOXA is precluded from accessing this regulatory element (Fig 7E). However, when SOX9 level declines and FOXA is elevated, FOXA2 competes off SOX9, accesses this binding site and activates *Col10a1* expression. The data are consistent with a model where FOXA2 and FOXA3 de-repress *Col10a1* by binding the regulatory regions bound by SOX9[22]. FOXA has been proposed to act as a pioneer factor to displace the linker histone and keep the enhancer accessible for specific TFs to activate gene expression in liver cells[88]. FOXA factors might initiate the hypertrophic program by out competing with repressive SOX factors to keep the chromatin accessible for other synergistic factors including RUNX2, MEF2C and SMAD1/4[22]. We surmise that SOX9 expression levels need to be lowered for this competition to take place as SOX9 forms a stable dimer at high concentrations that cannot be easily displaced. RUNX2 interaction with SOX9 depletes the effective level of SOX9 [89]. Furthermore, ZBTB20 and TRPS1, which are induced by SOX9, may repress SOX9 in the HZ in a negative-feedback loop [81, 90]. In view of the reported SOX9/AP1 cooperation in transactivating *Col10a1* expression, it is interesting to note that AP1 binding sites were also present close to the SOX9/FOXA binding motifs in the *Col10a1* enhancer[21]. This raises questions about whether FOXA2 could also cooperate with AP1 factors in promoting hypertrophy and if FOXA factors can compete to modulate the cooperative action of SOX9-AP1.

We have illustrated the powerful utility of integrating GP-DGEL with other databases as a discovery strategy to determine which biological processes and pathways, transcriptional regulators and their potential cooperating partners are active, in the growth plate. Our phasic GRN featuring SOX9, GLI and FOXA represents an initial template for the construction of a more complete model of chondrocyte differentiation that should incorporate a more complete set of TFs. In particular, it would be important to understand how SOX9-FOXA competition integrates with the SOX9-AP1 cooperation in promoting hypertrophy[21]. The protein interactome, non-coding RNAs, epigenetic status and chromatin/super-enhancer organization should also be taken into account in the future. It would be too great a task for a single study to investigate and functionally validate all the different target genes and processes identified. Therefore the gained information is shared as a public resource to facilitate and inspire additional discovery (S4 Fig). Mining and integration of the information in GP-DGEL with other emerging genome-wide data on the binding profiles of other transcription factors will be essential to extend our understanding of the complex and dynamic GRN mediating the transition steps in chondrocyte differentiation.

## Materials and methods

### Animals and growth plate fractionation

Transverse sections of the proximal tibia of 10-day-old female F1 (offspring of CBA and C57BL/6) mouse were obtained from chondrocyte sub-populations by cryosectioning. 5-micron sections were prepared and pooled into fractions consisting of 10 sections per fraction to ensure separation of each cell type in the growth plate. Samples were dissolved in Trizol reagent (Invitrogen) for RNA extraction. To guide the sub-division of chondrocyte populations into zones, every 10^th^ section was examined histologically and 10% of RNA was isolated from selected sections at regular intervals for RT-PCR analyses of known growth plate markers (S1C Fig), to guide the sub-division of chondrocyte populations into zones. Sox9 heterozygous null (*Sox9*^*+/-*^) mutants were generated by crossing *Sox9-flox* mice (gift of Andreas Schdel)[10] with *Protamine-Cre* transgenic mice (gift of Yelena Marchuk) [91]. Gli2 null (*Gli2*^*-/-*^) mice were a gift from C.C Hui[92].

### RNA isolation, reverse-transcription PCR and quantitative PCR

Total RNA was isolated according to the instructions for RNA isolation (Invitrogen). Prior to microarray analysis, 50ng total RNA was used to generate cDNA from each fraction by reverse transcription using SuperScript II reverse transcriptase and random hexamers. Semi-quantitative PCR analysis was performed to detect the expression of chondrogenic markers to identify the subpopulations of chondrocytes. Quantitative PCR was performed using Syber-Green master mixture to compare the expression levels of SOX9 target genes in the chondrocytes of wild type and Sox9^+/-^ mice.

### Microarray hybridization and data analysis

RNA quality and integrity were analyzed using RNA 6000 Nano Kit (Agilent). The pooled RNA was amplified for one cycle using MessageAmp^TM^ II-Biotin Enhanced Kit, then labeled and hybridized to Mouse Genome 430 2.0 GeneChip containing 45101 probe sets (Affymetrix) in the Centre for Genomic Sciences (the University of Hong Kong). All primary microarray data are deposited in the GEO website (GSE99306). Gene expression data for each zone in triplicate were normalized by using RMA algorithm in BioConductor software package [93]. The *k*-Means Clustering algorithm [94, 95] and Eisen software tools [96] were used to identify the distinct expression patterns of genes with Coefficient of Standard Deviation (C.S.D.) > 0.15 across 4 zones. For each gene, the C.S.D. value was calculated with the formula:

$C.S.D.=S/\bar{X}$, where S is the standard deviation and $\bar{X}$ is the mean expression level of the samples over the 4 zones. The Gene Ontology analysis was performed for each cluster of genes by using the Gene Ontology database [97] and the David Web Tools [98]. To identify differentiation phase-specific genes and differential patterns of expression across different zones, we defined a set of “On” and “Off” genes in the dataset. *Sox9* mRNA is expressed in the PZ and PHZ and is down-regulated in UHZ and LHZ. In contrast, *Col10a1* is exclusively expressed in PHCs and HCs during hypertrophic differentiation in the PHZ, UHZ and LHZ. Therefore, we used the expression level of *Sox9* in HZ (356, the average from UHZ and LHZ) and that of *Col10a1* in PZ (511) to set the threshold of “On-Off” states for each zone (S2A Table).

### DME motif discovery

The DME analysis were performed by using the Software package CREAD [99] with input from the promoter sequences extracted from 1k upstream and 200 bp downstream of TSS of the genes in each cluster. The background sequence file was generated by using the computer program ‘fasta-shuffle-notryptic.pl’ in the Bioinformatics CPAN Perl module of InSilicoSpectro-Databanks version 0.0.43. The Matcompare program in the CREAD package was used to compute the similarity between the identified DME motif and those in the TRANSFAC, JASPAR and ENCODE databases [100–103]. The Position Frequency Matrices and the consensus DNA binding sequences of the transcription factors were compiled from TRANSFAC database and the literature. Foreground (FG) represents the number of occurrences of the identified DNA motifs in the set of promoter DNA sequences. Background (BG) represents the number of occurrences of the motifs in the randomly generated DNA sequences. The ratio of FG/BG indicates the fold enrichment of the identified motifs in the foreground over the background set of sequences [104].

### MEME dimer motif enrichment analyses

Position Weight Matrix identified in DME promoter analyses and the functional SOX9_COL2C1, COL2C2 [24], COL2C3 [65] binding consensus (S7 Table) were utilized as the seed motif for screening of SOX9 monomer binding sites in the genomic region within 10kb from TSS of the zone DEGs. The DNA sequences of 25-bp flanking the identified SOX9 monomer site on both sides were retrieved from Reference Genome (mm9) after removing DNA repeats. The MEME program was run with the command:

$meme monomer_site_flanking_sequence.fq -dna -mod anr -nmotifs 2 -w 30 -oc meme_out_30bp -pal

The parameter on motif length was set to range from 10 to 30-bp with the palindrome search mode activated.

### Screening of evolutionarily conserved transcription factor binding element

The genomic sequences of evolutionarily conserved non-coding elements in the promoter and intergenic regions of each gene were retrieved from the Mouse Reference Genome Sequence of NCBI build 37, mm9. The conservation scores of DNA sequences for 30-vertebrates and the genomic coordinates of the non-coding elements were obtained from UCSC Genome Browser Database [105]. The algorithm [106] was implemented to match the Position Weight Matrix of the transcription factors with the genomic DNA sequences for screening of their binding elements. A match between the TF and the target sequence was accepted if the sequence similarity score was over 85% and the UCSC phastCons DNA conservation score was over 300. For prediction of SOX9 COL2C1, COL2C2 and COL2C3 binding elements, we searched for the exact matches of the binding motifs in the sequence of evolutionarily conserved non-coding DNA elements. The Position Weight Matrices used for identification of SOX9 dimer and GLI binding elements were constructed from the SOX9 binding HMG core sequence and the GLI binding consensus in TRANSFAC database respectively.

### SOX9 ChIP-seq and GLI ChIP-chip peak detection

Detailed methods of rib chondrocyte isolation and SOX9 ChIP-seq experiment were described as reported [33]. The GLI1 promoter and GLI3 Genome-Wide ChIP-chip datasets were downloaded from GEO database (GSE11062 and GSE11063)[73] and converted from mm8 to mm9 assembly by using the UCSC Toolkit [107]. The SOX9 and GLI binding regions were identified by applying the procedure for local maxima finding [108] with 25- and 50-bp genomic windows respectively.

### Fisher’s exact test and odds ratio calculation

Fisher’s exact test on two-tailed P value was performed for a 2x2 contingency table with GraphPad Software (GraphPad Software, Inc.), where group 1 represents the PZ, PHZ and HZ DEGs in the clustering heatmap, and group 2 represents the DEGs containing SGBR. The Odds Ratio number was calculated with the formula,

$$Odds\;Ratio=\frac{{PZ,PHZ}_{SGBR}/{HZ}_{SGBR}}{{PZ,PHZ}_{total}/{HZ}_{total}}$$

where PZ,PHZ_SGBR_ is the number of SOX9/GLI common target in the PZ, PHZ gene sets, and PZ,PHZ_total_ is the total number of the PZ, PHZ genes in the heatmap. Fisher’s Exact Test was performed with: (i) varied inter-peak distances between SOX9 and GLI binding regions; (ii) varied genomic distance between SGBRs and the target gene TSS as in previous studies[109, 110]. The intergenic region was defined by the two nearest genes located at the 5’-end and 3’-end of the gene in query. The gene annotation information was downloaded from UCSC Genome Database.

### In situ hybridization

In situ hybridization was performed as previously described[111]. Hind limbs dissected from 10-day-old F1 littermates were fixed in 4% paraformaldehyde overnight at 4°C and decalcified in 0.5M EDTA for 24h before embedding in paraffin. [^35^S]UTP labeled probes for the selected genes were hybridized on the paraffin sections of the hind limbs.

### Immunofluorescence

The paraffin sections were dewaxed and rehydrated. For cryosection, tissues were fixed in 4% PFA overnight before immersed in 30% sucrose. Sections were blocked with blocking buffer (5% BSA or goat serum, 0.5% Tween20) for 1 hour at room temperature. The primary antibodies of rabbit anti-Foxp2 (1:400; Abcam), rabbit anti-SOX9 (1:500, Millipore), guinea pig anti-SOX9 (1:2000, gift from V. Lee, STEMCELL Technologies) and rabbit anti-FOXA2 (1:500, Millipore) were diluted in blocking buffer and applied on the sections at 4°C overnight. The signal was visualized by using 1:500 goat-anti-rabbit or donkey-anti-guinea pig antibodies and mounting with Vectashield® mounting medium containing DAPI.

### Electrophoretic mobility shift assay

EMSAs were performed as described [66]. DNA probes were prepared with cy5-label at the 5’ end of the forward strand and reverse strand unlabeled. Equimolar amounts of complementary strands were annealed at 95°C for 5 min and subsequent cooling to 4°C at 1°C /min. Reaction mixtures (60nM probes and varying concentrations of proteins) were incubated at 4°C in the dark for 4h and electrophoresed at 200V for ~40min at 4°C in the dark. The gels were imaged with a Typhoon FLA-7000 PhosphorImager (FUJIFILM).

### Cell culture, DNA transfection and luciferase assay

ATDC5 cells were grown in DMEM/F12 supplemented with 5% fetal bovine serum, human transferrin (10μg/ml) and sodium selenite (5ng/ml), and seeded in 12-well plates. With ~70%–80% confluency on the following day, the cells were transiently transfected with pGL3-basic luciferase reporters containing different regulatory elements using Lipofectamine 2000 (Invitrogen). Luciferase activity was measured using the Dual luciferase reporter assay kit (Promega) according to the manufacturer's instructions. Luciferase expression is given as a fold-change relative to the activity of *Renilla* luciferase.

### Ethics statement

The work with the use of mice and their care was approved in accordance with our institutional guidelines (Committee for the Use of Live Animals in Research, the University of Hong Kong).

## Supporting information

S1 Fig Fractionation of different chondrocyte populations in wild type growth plate for transcriptomic profiling. (A) A schematic diagram of tibia growth plate showing the expression patterns of chondrogenic markers (*Col2a1*, *Col10a1*, *Ihh*, *Ppr*, *Sox9* and *Mmp13*) and the rationale to fractionate the growth plate into different chondrocyte populations. (B) Histology of chondrocytes at different differentiation stages. The section between each fraction was stained with Safranin O showing the distinctive morphology of PCs, PHCs, HCs and trabecular bone (TB). (C) Expression profiles of characteristic chondrogenic markers in WT growth plate. Fractions from PZ to HZ of growth plate based on the histological morphologies were chosen for RNA extraction. cDNA was synthesized using 10% of the total RNA from each fraction and analyzed by Semi-quantitative PCR on the expression profiles of chondrogenic markers. (D) Flowchart for the data processing, motif analyses, validation and model generation. (E) The numbers of genes that were transcriptionally turned ‘On’ in one zone but ‘Off’ in the others as determined by using the expression level of Col10a1 in PZ and Sox9 in HZ as threshold were shown in the Venn chart.
(PPTX)

S2 Fig Validation of predicted SOX9 binding sites on target genes. (A-C) Predicted SOX9 binding sites, SOX9 binding regions and conservation score were shown in the loci of *Cyr61*, *Trps1* and *Ptch1*. The inverted triangles (orange) indicate the predicted SOX9 binding sites selected for validation. BS: binding site; BR: binding region. (D) Predicted SOX9 binding sites in the promoter of *Cyr61* and *Ptch1*, and intron I of *Trps1* were validated by ChIP assay using E13.5 limb chondrocytes. The ChIP-qPCR indicated the SOX9 interaction with the predicted binding sites on *Cyr61*, *Trps1* and *Ptch1*. SOX9 binding site on *Acan* promoter was used as a positive control.
(PPTX)

S3 Fig Expression patterns of *Sox9* and *Gli1* in the growth plate. (A-B) *In vivo* expression patterns of *Gli1* (A) and *Sox9* (B) were shown by in situ hybridization. The PZ, PHZ and HZ were separated by the white-dot lines. (C-E) Significant upregulation of SOX9, *Gli1* and *Gli2* in ATDC5 cells, revealed by western blot (C) and quantitative RT-PCR (D, E). (F) GLI1 activated the *Ptch1* reporter in transiently transfected ATDC5 cells.
(PPTX)

S4 Fig GP-DGEL: A searchable online Gene Expression Library for gene discovery. (A) A Growth Plate Differential Gene Expression Library (GP-DGEL) was generated for the query of gene expression pattern changes in the growth plate and their associated gene regulatory regions targeted by SOX9, GLI1 and GLI3 transcription factors.
(PPTX)

S1 Table Genes showing ‘On’-‘Off’ expression in the growth plate. (DOCX)

S2 Table Differentially expressed genes (DEGs) and their association with skeletal phenotypes. **(**A) DEGs in PZ, PHZ, UHZ and LHZ and their association with mouse and human abnormalities in skeletal system. Genes with the Coefficient of Standard Deviation (CSD) of expression levels among the four zones greater than 0.15 are defined as the DEGs. (B) DEGs associated with mouse skeletal phenotypes (396 genes). '-' represents the references from International Mouse Phenotyping Consortium or the direct submissions from third party organizations. (C) DEGs associated with human skeletal diseases in orthologous (93 genes).
(XLSX)

S3 Table Enriched biological process in the growth plate. (DOCX)

S4 Table Enriched signaling pathways in the growth plate. Genes involved in IHH, IGF, WNT, BMP, FGF, HIPPO and NOTCH signaling pathways are listed. Fisher’s exact probability test was performed by categorizing the genes into the 2 groups (PZ and PHZ versus UHZ and LHZ). The whole set of the DEGs in S2 Table was set as the background group.
(DOCX)

S5 Table Differentially expressed transcription factors and their association with skeletal phenotypes. The association of these TFs with human skeletal diseases and mouse skeletal phenotypes according to the OMIM and MGI databases are also summarized respectively. CSD: coefficient of standard deviation (mean normalized standard deviation); Exp: expression level; H: human skeletal disorders; M: mouse skeletal phenotypes; 1: mutations of the gene are associated with H or M; 0: mutations of the gene are not associated with H or M; -: mutations of the gene have not been reported to be associated with H or M.
(DOCX)

S6 Table Enriched SOX9 dimer motifs in PZ and PHZ MEME program was applied to identify SOX9 dimer motifs of varied width (W) ranging from 20 to 50-bp and P-value<1.0e^-3^ was set as the threshold for statistical significance. We defined the spacer length L_*n*_ as the number of DNA bases located between the paired SOX9 core binding consensus, represented as 5’-AACAA(L_*n*_)-TTGTT3’.
(DOCX)

S7 Table Predicted SOX9 and GLI motif pattern nearby SOX9 peaks. The SOX9 monomer (SOX9 COL2C1, COL2C2, COL2C3 and consensus from Transfac database), dimer binding sites and GLI binding sites were screened in the conserved noncoding DNA elements for DEGs. (A) Numbers of SOX9 and GLI motifs nearby SOX9 peaks in DEGs. (B) Summary of SOX9 and GLI motifs nearby SOX9 peaks in DEGs. (C to G) Prediction of SOX9 monomer and dimer binding sites in DEGs. (H) Prediction of GLI motifs nearby SOX9 peaks in DEGs.
(XLSX)

S8 Table SOX9, GLI1, GLI3 and FOXA2 ChIP-seq peaks in the target genes. **(**A1) Genome wide SOX9 peaks associated with the nearest genes; 177890 associations (p-value <0.05, peak height>5) were detected. Among these, the top 72769 associations with 36076 distinct peaks identified in 21083 genes (p-value < 0.05, peak height>15) were selected for further analysis. (A2) Number of SOX9 peaks in each target gene (peak height > 15, p-value<0.05). (B1) SOX9 peaks in DEGs (p-value<0.05, peak height > 15). (B2) Number of SOX9 peaks in each DEG. S8C1 GLI1 peaks (promoter) associated with the nearest genes. Totally 7648 distinct peaks associated with 9159 distinct genes were detected. (C2) Number of GLI1 peaks in each target gene. (D1) GLI1 peaks in DEGs. (D2) Number of GLI1 peaks in each DEG. (E1) Genome-wide GLI3 peaks associated with the nearest genes. Totally 58250 distinct peaks associated with 18990 genes were detected. (E2) Number of GLI3 peaks in each target gene. (F1) GLI3 peaks in DEGs. (F2) Number of GLI3 peaks in each DEG. (G) FOXA2 binding peaks in *Sox9* gene.
(XLSX)

S9 Table SOX9 binding sites identified and validated in *Cyr61*, *Trps1* and *Ptch1*. Conserved SOX9 binding sites in the regulatory elements of *Cyr61*, *Trps1* and *Ptch1* were selected for ChIP-PCR validation. The genomic coordinates of these SOX9 binding site and their relative position to the nearest SOX9, GLI1 and GL3 binding peaks were shown.
(DOCX)

S10 Table Fisher exact test on SOX9-GLI common targets. **(**A1) Association between SOX9-GLI inter-peak distance and target gene expression patterns (PZ, PHZ, UHZ over LHZ genes). (A2) Association between SOX9-GLI inter-peak distance and target gene expression patterns, with varied distance of SGBR from TSS. (A3) Association between SGBR distance-to-TSS and target gene expression patterns (PZ, PHZ, UHZ genes versus LHZ genes). (B1) Odds Ratios for GBR, SBR and SGBR (50-bp inter-peak distance) target genes zone specifically expressed in (PZ, PHZ, UHZ) over LHZ. (B2) Fisher's exact on SOX9 target genes (SBR) in zone specific expression. (B3) Fisher's exact test on GLI1 target genes. (B4) Fisher's exact test on GLI3 target genes. (C1) Genomic locations of SGBRs in DEGs (inter-peak distance < 50-bp, intergenic region >25kb). (C2) Number of SGBRs in each DEG. (D1) Genomic locations and numbers of SGBRs in DEGs (inter-peak distance < 250 bp, intergenic region >25kb). (D2) Number of SGBRs in each DEG.
(XLSX)
