# Supplementary material for: Synergistic co-regulation and competition by a SOX9-GLI-FOXA phasic transcriptional network coordinate chondrocyte differentiation transitions
Source: PLoS Genet. 2018 Apr 16;14(4):e1007346. doi: 10.1371/journal.pgen.1007346 (PMC5919691; doi:10.1371/journal.pgen.1007346)
Supplement: S1 Fig — (A) A schematic diagram of tibia growth plate showing the expression patterns of chondrogenic markers (Col2a1, Col10a1, Ihh, Ppr, Sox9 and Mmp13) and the rationale to fractionate the growth plate into different chondrocyte populations. (B) Histology of chondrocytes at different differentiation stages. The section between each fraction was stained with Safranin O showing the distinctive morphology of PCs, PHCs, HCs and trabecular bone (TB). (C) Expression profiles of characteristic chondrogenic markers in WT growth plate. Fractions from PZ to HZ of growth plate based on the histological morphologies were chosen for RNA extraction. cDNA was synthesized using 10% of the total RNA from each fraction and analyzed by Semi-quantitative PCR on the expression profiles of chondrogenic markers. (D) Flowchart for the data processing, motif analyses, validation and model generation. (E) The numbers of genes that were transcriptionally turned ‘On’ in one zone but ‘Off’ in the others as determined by using the expression level of Col10a1 in PZ and Sox9 in HZ as threshold were shown in the Venn chart. (PPTX) [file pgen.1007346.s001.pptx]

## Slide 1
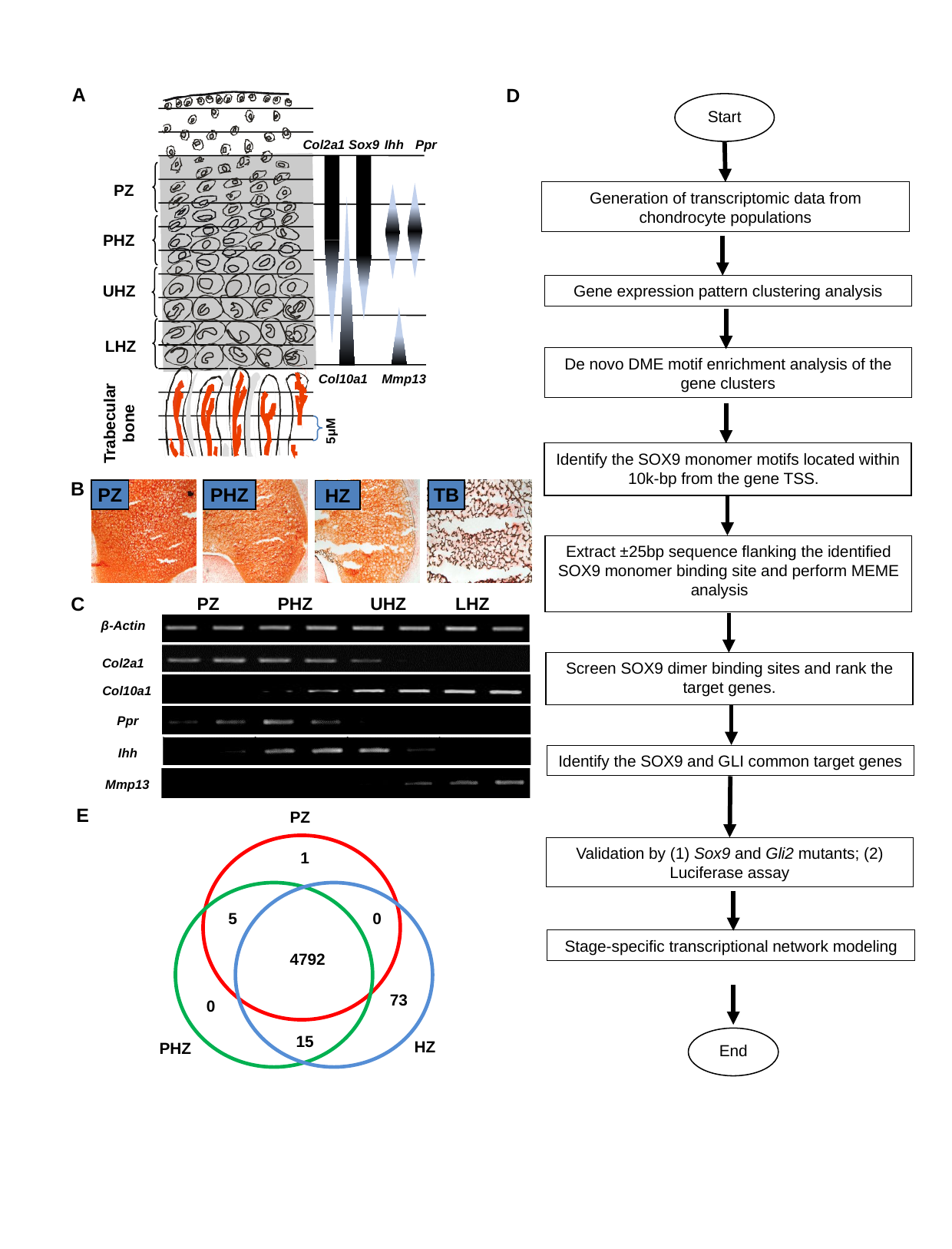

A
Col2a1
Ihh
Ppr
Sox9
Col10a1
Mmp13
PZ
PHZ
UHZ
LHZ
Trabecular bone
5μM
D
Start
Generation of transcriptomic data from chondrocyte populations
Gene expression pattern clustering analysis
De novo DME motif enrichment analysis of the gene clusters
Identify the SOX9 monomer motifs located within 10k-bp from the gene TSS.
Extract ±25bp sequence flanking the identified SOX9 monomer binding site and perform MEME analysis
Screen SOX9 dimer binding sites and rank the target genes.
Identify the SOX9 and GLI common target genes
Validation by (1) Sox9 and Gli2 mutants; (2) Luciferase assay
Stage-specific transcriptional network modeling
End
B
PZ
PHZ
TB
HZ
C
PZ
PHZ
UHZ
LHZ
β-Actin
Col2a1
Col10a1
Ppr
Ihh
Mmp13
E
PZ
1
5
0
4792
73
0
15
HZ
PHZ
