# Supplementary material for: Synergistic co-regulation and competition by a SOX9-GLI-FOXA phasic transcriptional network coordinate chondrocyte differentiation transitions
Source: PLoS Genet. 2018 Apr 16;14(4):e1007346. doi: 10.1371/journal.pgen.1007346 (PMC5919691; doi:10.1371/journal.pgen.1007346)
Supplement: S2 Fig — (A-C) Predicted SOX9 binding sites, SOX9 binding regions and conservation score were shown in the loci of Cyr61, Trps1 and Ptch1. The inverted triangles (orange) indicate the predicted SOX9 binding sites selected for validation. BS: binding site; BR: binding region. (D) Predicted SOX9 binding sites in the promoter of Cyr61 and Ptch1, and intron I of Trps1 were validated by ChIP assay using E13.5 limb chondrocytes. The ChIP-qPCR indicated the SOX9 interaction with the predicted binding sites on Cyr61, Trps1 and Ptch1. SOX9 binding site on Acan promoter was used as a positive control. (PPTX) [file pgen.1007346.s002.pptx]

## Slide 1
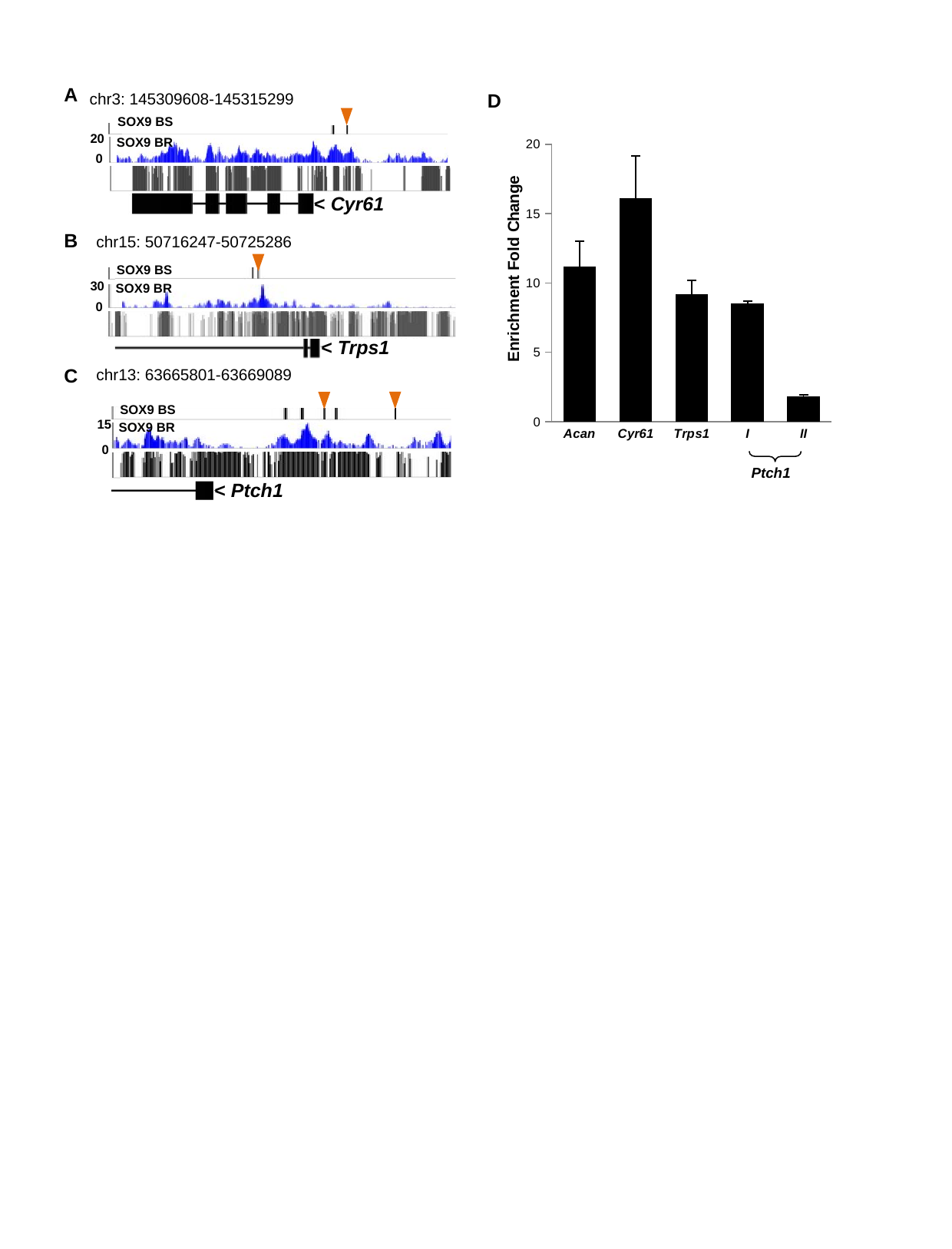

A
chr3: 145309608-145315299
D
SOX9 BS
20
SOX9 BR
0
< Cyr61
### Chart
| Category | Sox9 |
|---|---|
| Acan | 11.18768563518483 |
| Cyr61 | 16.12558639967088 |
| Trps1 | 9.176254590352377 |
| I | 8.537464777628573 |
| II | 1.799653371175124 |B
chr15: 50716247-50725286
SOX9 BS
30
SOX9 BR
0
< Trps1
C
chr13: 63665801-63669089
SOX9 BS
15
SOX9 BR
0
< Ptch1
Ptch1
