# Supplementary material for: Synergistic co-regulation and competition by a SOX9-GLI-FOXA phasic transcriptional network coordinate chondrocyte differentiation transitions
Source: PLoS Genet. 2018 Apr 16;14(4):e1007346. doi: 10.1371/journal.pgen.1007346 (PMC5919691; doi:10.1371/journal.pgen.1007346)
Supplement: S3 Fig — (A-B) In vivo expression patterns of Gli1 (A) and Sox9 (B) were shown by in situ hybridization. The PZ, PHZ and HZ were separated by the white-dot lines. (C-E) Significant upregulation of SOX9, Gli1 and Gli2 in ATDC5 cells, revealed by western blot (C) and quantitative RT-PCR (D, E). (F) GLI1 activated the Ptch1 reporter in transiently transfected ATDC5 cells. (PPTX) [file pgen.1007346.s003.pptx]

## Slide 1
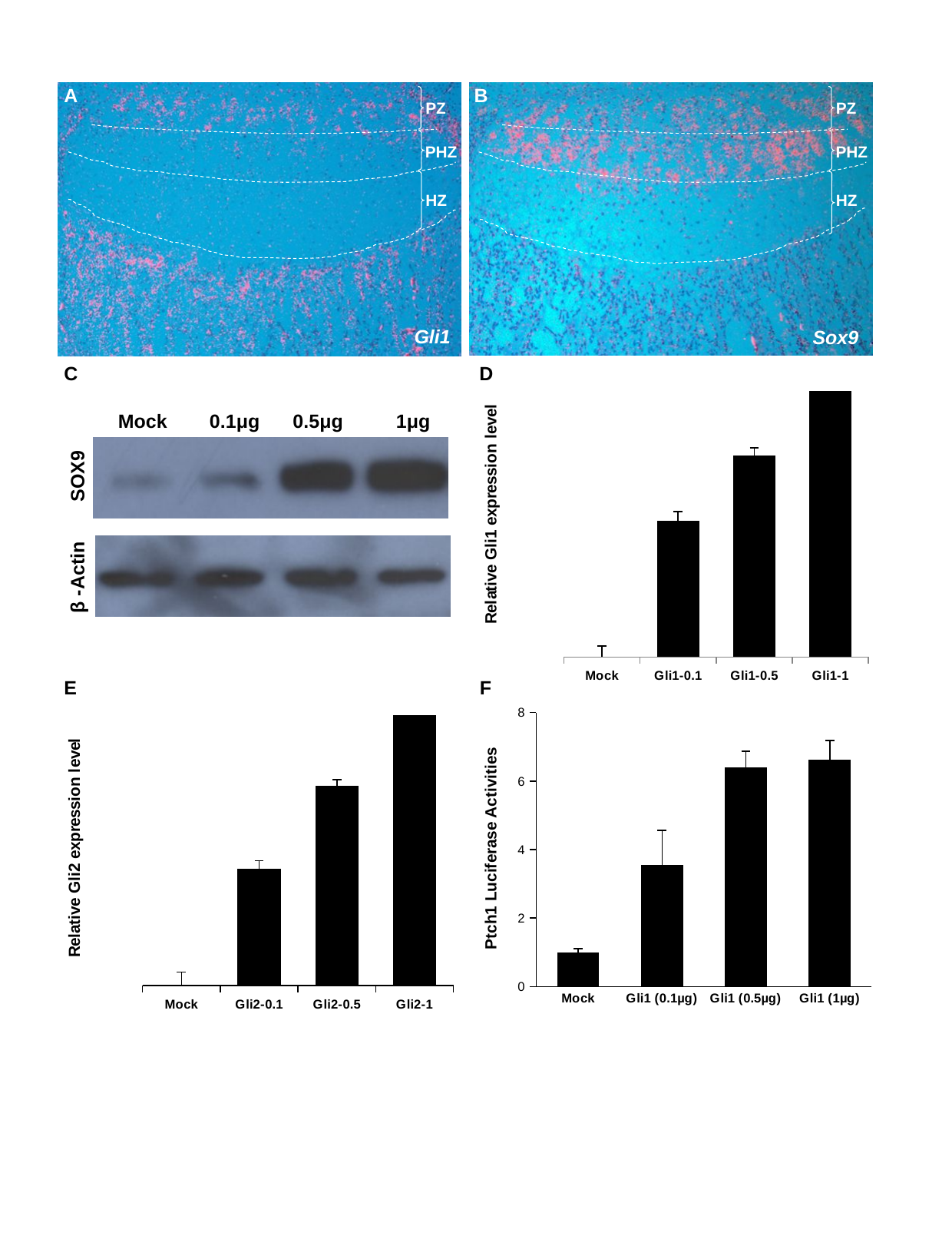

B
A
PZ
PHZ
HZ
Gli1
PZ
PHZ
HZ
Sox9
C
D
### Chart
| Category | Relative Gli1 level |
|---|---|
| Mock | 2.0064210426796976 |
| Gli1-0.1 | 315.7305788628177 |
| Gli1-0.5 | 3651.612159253871 |
| Gli1-1 | 40088.03088110671 |Mock
0.1μg
0.5μg
1μg
SOX9
β -Actin
E
F
### Chart
| Category | Relative Gli2 level |
|---|---|
| Mock | 1.549116506546502 |
| Gli2-0.1 | 167.35418041011224 |
| Gli2-0.5 | 4680.645208921839 |
| Gli2-1 | 80224.39773075937 |
### Chart
| Category | Normalized activities |
|---|---|
| Mock | 1.0 |
| Gli1 (0.1µg) | 3.560627291272221 |
| Gli1 (0.5µg) | 6.39633668710917 |
| Gli1 (1µg) | 6.627006315717919 |Ptch1 Luciferase Activities
