# Supplementary figures and images for: Synergistic co-regulation and competition by a SOX9-GLI-FOXA phasic transcriptional network coordinate chondrocyte differentiation transitions
Source: PLoS Genet. 2018 Apr 16;14(4):e1007346. doi: 10.1371/journal.pgen.1007346 (PMC5919691; doi:10.1371/journal.pgen.1007346)

## Slide 1
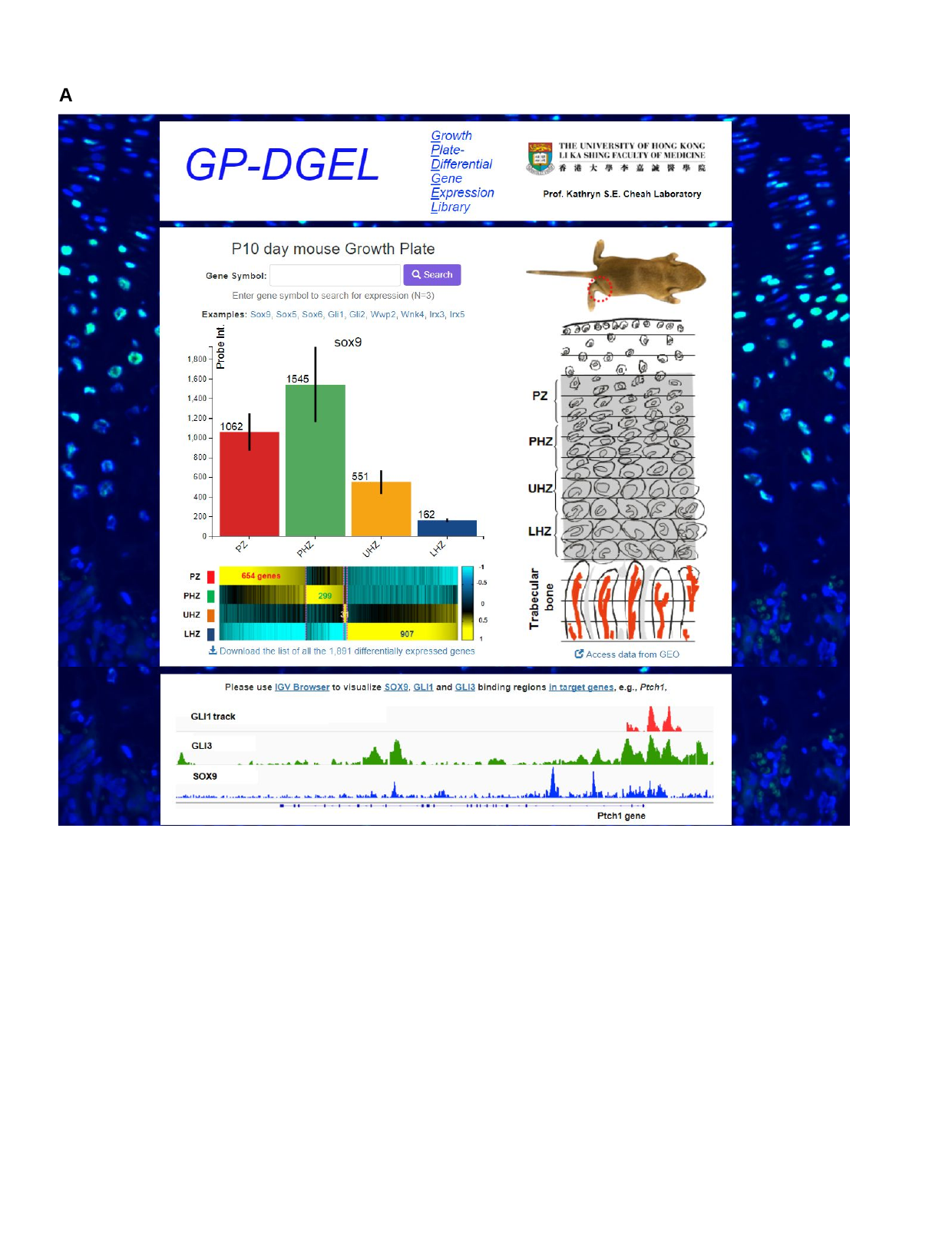

A

Supplement: S4 Fig — (A) A Growth Plate Differential Gene Expression Library (GP-DGEL) was generated for the query of gene expression pattern changes in the growth plate and their associated gene regulatory regions targeted by SOX9, GLI1 and GLI3 transcription factors. (PPTX) [file pgen.1007346.s004.pptx]
