# Supplementary material for: Synergistic co-regulation and competition by a SOX9-GLI-FOXA phasic transcriptional network coordinate chondrocyte differentiation transitions
Source: PLoS Genet. 2018 Apr 16;14(4):e1007346. doi: 10.1371/journal.pgen.1007346 (PMC5919691; doi:10.1371/journal.pgen.1007346)
Supplement: S1 Table — (DOCX) [file pgen.1007346.s005.docx]

| **Table S1 Genes showing ‘On’-‘Off’ expression in the growth plate.** | |
| --- | --- |
| **Symbol** | **Gene description** |
| **PZ-‘On’ and PHZ, HZ-‘Off’** | |
| *Fzd9* | Frizzled homolog 9 (Drosophila) |
| **PZ, PHZ-‘On’ and HZ-‘Off’** | |
| *Gfpt2* | Glutamine fructose-6-phosphate transaminase 2 |
| *Glt25d2* | Glycosyltransferase 25 domain containing 2 |
| *S100b* | S100 protein, beta polypeptide, neural |
| *Sox9* | SRY-box containing gene 9 |
| *Syt8* | synaptotagmin VIII |
| **PZ-‘Off’ and PHZ,HZ-‘On’** | |
| *Aldh1a3* | Aldehyde dehydrogenase family 1 member A3 |
| *Bmp2* | Bone morphogenetic protein 2 |
| *Col10a1* | Collagen alpha-1(X) chain |
| *Evi2a* | Protein EVI2A |
| *Fat3* | Protocadherin Fat 3 |
| *Hebp2* | Heme-binding protein 2 |
| *Hmgcr* | 3-hydroxy-3-methylglutaryl-coenzyme A reductase |
| *Irx5* | Iroquois-class homeodomain protein IRX-5 |
| *Lrp4* | Low-density lipoprotein receptor-related protein 4 |
| *Pawr* | PRKC apoptosis WT1 regulator protein |
| *Phex* | Metalloendopeptidase homolog PEX |
| *Ptprz1* | protein tyrosine phosphatase, receptor type Z, polypeptide 1 |
| *Rab27b* | Ras-related protein Rab-27B |
| *Slc36a2* | Proton-coupled amino acid transporter 2 |
| *Slc38a4* | Sodium-coupled neutral amino acid transporter 4 |
| **PZ, PHZ ‘Off’ and HZ ‘On’** | |
| *Agpat3* | 1-acyl-sn-glycerol-3-phosphate acyltransferase gamma |
| *Aplp1* | Amyloid-like protein 1 |
| *Aurka* | Serine/threonine-protein kinase 6 |
| *BC028528* | Uncharacterized protein C1orf54 homolog |
| *Carhsp1* | Calcium-regulated heat stable protein 1 |
| *Ccnb1* | G2/mitotic-specific cyclin-B1 |
| *Cdc42ep2* | Cdc42 effector protein 2 |
| *Cdk2ap2* | Cyclin-dependent kinase 2-associated protein 2 |
| *Centd3* | Arf-GAP with Rho-GAP domain, ANK repeat and PH domain-containing protein 3 |
| *Cfh* | Complement factor H |
| *Commd6* | COMM domain-containing protein 6 |
| *D16H22S680E* | Ser/Thr-rich protein T10 in DGCR region |
| *Ddx59* | Probable ATP-dependent RNA helicase DDX59 |
| *Dhrs7b* | Dehydrogenase/reductase SDR family member 7B |
| *Egr3* | Early growth response protein 3 |
| *Eif2ak1* | Eukaryotic translation initiation factor 2-alpha kinase 1 |
| *Enpp6* | Ectonucleotide pyrophosphatase/phosphodiesterase family member 6 |
| *Fes* | Proto-oncogene tyrosine-protein kinase Fes/Fps |
| *Fgd5* | FYVE, RhoGEF and PH domain-containing protein 5 |
| *Gimap6* | GTPase IMAP family member 6 |
| *Gipc1* | PDZ domain-containing protein GIPC1 |
| *Glb1* | Beta-galactosidase |
| *Glrx* | Glutaredoxin-1 |
| *Gnptab* | N-acetylglucosamine-1-phosphotransferase subunits alpha/beta |
| *Gpd1l* | Glycerol-3-phosphate dehydrogenase 1-like protein |
| *Gprc5c* | G-protein coupled receptor family C group 5 member C |
| *Gucy1a3* | Guanylate cyclase soluble subunit alpha-3 |
| *Hat1* | Histone acetyltransferase type B catalytic subunit |
| *Hes1* | Transcription factor HES-1 |
| *Ibrdc3* | E3 ubiquitin-protein ligase RNF19B |
| *Icam2* | Intercellular adhesion molecule 2 |
| *Irx3* | Iroquois-class homeodomain protein IRX-3 |
| *Itga4* | Integrin alpha-4 |
| *Kti12* | Protein KTI12 homolog |
| *LOC620893* | Polypeptide N-acetylgalactosaminyltransferase 3 |
| *Mcoln1* | Mucolipin-1 |
| *Megf10* | Multiple epidermal growth factor-like domains 10 |
| *Mical3* | Uncharacterized protein KIAA0819 |
| *Mmp16* | Matrix metalloproteinase-16 |
| *Mxd1* | MAD protein |
| *Ncf4* | Neutrophil cytosol factor 4 |
| *Nhsl1* | NHS-like protein 1 |
| *Pitpnm1* | Membrane-associated phosphatidylinositol transfer protein 1 |
| *Ppm1e* | Protein phosphatase 1E |
| *Prkx* | Serine/threonine-protein kinase PRKX |
| *Prps2* | Ribose-phosphate pyrophosphokinase 2 |
| *Psme3* | Proteasome activator complex subunit 3 |
| *Ptgs2* | Prostaglandin G/H synthase 2 |
| *Rad23a* | UV excision repair protein RAD23 homolog A |
| *Ralgps2* | Ras-specific guanine nucleotide-releasing factor RalGPS2 |
| *Rapgef5* | Rap guanine nucleotide exchange factor 5 |
| *Rbp7* | Retinoid-binding protein 7 |
| *Rhod* | Rho-related GTP-binding protein RhoD |
| *Sema5a* | Semaphorin-5A |
| *Serpina3g* | Serine protease inhibitor A3G |
| *Sh2b3* | SH2B adapter protein 3 |
| *Sipa1* | Signal-induced proliferation-associated protein 1 |
| *Sirpa* | Tyrosine-protein phosphatase non-receptor type substrate 1 |
| *Slc43a1* | Large neutral amino acids transporter small subunit 3 |
| *Smad6* | Mothers against decapentaplegic homolog 6 |
| *Smap1l* | Stromal membrane-associated protein 2 |
| *Spata13* | Spermatogenesis-associated protein 13 |
| *Src* | Neuronal proto-oncogene tyrosine-protein kinase Src |
| *St3gal1* | CMP-N-acetylneuraminate-beta-galactosamide-alpha-2,3-sialyltransferase |
| *T2bp* | TRAF-interacting protein with FHA domain-containing protein A |
| *Tie1* | Tyrosine-protein kinase receptor Tie-1 |
| *Tmc6* | Transmembrane channel-like protein 6 |
| *Tmem120a* | Transmembrane protein 120A |
| *Tpx2* | TPX2, microtubule-associated protein homolog |
| *Trp53i11* | transformation related protein 53 inducible protein 11 |
| *Vamp5* | Vesicle-associated membrane protein 5 |
| *Zeb2* | Zinc finger E-box-binding homeobox 2 |
| *Zfp711* | zinc finger protein 711 |
