# Supplementary material for: Synergistic co-regulation and competition by a SOX9-GLI-FOXA phasic transcriptional network coordinate chondrocyte differentiation transitions
Source: PLoS Genet. 2018 Apr 16;14(4):e1007346. doi: 10.1371/journal.pgen.1007346 (PMC5919691; doi:10.1371/journal.pgen.1007346)
Supplement: S3 Table — (DOCX) [file pgen.1007346.s007.docx]

| **Table S3 Enriched Biological Processes in each zone.** | | |
| --- | --- | --- |
| **Biological Processes** | **P-Value** | **Genes** |
| **PZ** | | |
| **Skeletal system development** | **1.16E-12** | *Fgfr2, Thra, Pdlim7, Tgfb3, Gabbr1, Col2a1, Sox6, Gli2, Hoxc4, Chst11, Ankrd11, Hoxa10, Pkd1, Hoxa9, Col11a2, Mn1, Pitx1, Idua, Prkca, Tbx15, Rarg, Hspg2, Igf1, Igf2, Npr2, Gas1, Wwtr1, Hoxc10, Shox2, Acvr2a, Tulp3, Sost, Ror2, Mapk8, Igfbp5* |
| **Regulation of smoothened signaling pathway** | **2.57E-05** | *Shox2, Fgfr2, Gpc3, Tulp3, Ptch1, Gas1, Gli2* |
| **Regulation of transcription** | **2.01E-04** | *Aebp1, Thra, Lmo4, Cnot3, Tgfb3, Gli2, Cbx6, Fubp1, Smarcd3, Sdpr, Hoxc4, Trak1, Creb3l2, Pitx1, Myst4, Zfp354c, Brd8, Zbtb20, Zfp692, Tbx15, Rarg, Ssbp2, Rcor3, Hdac10, Med12, Hmg20a, Hoxc10, Brwd1, Ptrf, Zfp462, Jun, Prdm5, Mdm4, Rbm39, Jmjd1c, Zfp260, Carm1, Ablim1, Bclaf1, Tshz2, Lcorl, Ndn, Nfix, Sox6, Nr3c1, Plagl1, Tsc22d3, Tspyl2, Hoxa10, Nfat5, Tef, Per1, Hoxa9, Limd1, Bcl9l, Nfatc4, Zfp521, Per3, Nfatc2, Runx3, Chd3, Srebf1, Maf, Jarid2, Lmcd1, Fzd1, Nr4a1, Igf1, Ilf3, Ski, 4930422i07rik, Wwtr1, Zfp101, Foxp1, Safb2, Foxp2, Shox2, Notch3, Dbp, Bnc2, Sfpq, Rbak, Zranb1, Tgfbr3, Phf21a, Fabp4, Nfic, Nfia, Ncor2, Rere, Nfib* |
| **Regulation of cell proliferation** | **3.31E-04** | *Fgfr2, Cav1, Il6st, Il18, Tgfb3, Timp2, Gli2, H19, Gpc3, Chst11, Runx3, Prkca, Rarg, Jarid2, Il11ra1, Igf1, Gas1, Foxp1, Foxp2, Shox2, Trim35, Dbp, Ptprv, Tial1, Jun, Fabp4, Tgfbr3, Ptch1, Mdm4, Mab21l2, Nfib* |
| **Positive regulation of cell adhesion** | **0.001** | *Smoc2, Fbln2, Egfl6, Nid1, Col8a1, Abi3bp, Cyr61* |
| **Regionalization** | **0.003** | *Rarg, Tcap, Ldb1, Gas1, Gli2, Hoxc10, Acvr2a, Tulp3, Hoxc4, Ift172, Hoxa10, Hoxa9, Ror2, Ptch1, Pofut1* |
| **Regulation of cell communication** | **0.005** | *Igf1r, Rgs11, Grb10, Lect1, Tnxb, S100b, Sipa1l1, Fgfrl1, C1qtnf2, Ntrk2, Rgs5, Ptch1* |
| **PHZ** | | |
| **Sterol metabolic process** | **1.15E-10** | *Cyp51, Soat1, Sc5d, Hdlbp, Ldlr, Hmgcr, Hmgcs1, Fdft1, Sc4mol, Dhcr7, Insig1, Scarb1, Idi1, Hsd17b7* |
| **Positive regulation of cell adhesion** | **3.69E-06** | *A930038c07rik, Smoc2, Npnt, Ccdc80, Edil3, Thbs1, Col8a1, Cd24a* |
| **Skeletal system development** | **0.001** | *Mef2c, Impad1, Lgals3, Prrx2, Sox6, Sox9, Ihh, Fgfr3, Tgfb2, Mef2d, Col9a1, Eya1, Insig1, Acan, Ptn, Bmp6* |
| **Cell motility** | **0.004** | *Ccdc88a, Ndn, Cspg4, Nr4a2, Amot, Tnn, Scarb1, Pafah1b1, Prkg1, Cd24a, Dclk1, Tgfb2* |
| **Regulation of kinase activity** | **0.008** | *Cav3, Map3k5, Ccdc88a, Hmgcr, Cspg4, Fabp4, Gadd45b, Cd24a, Lats2* |
| **Actin cytoskeleton organization** | **0.013** | *Pfn2, Nisch, Nrap, Neb, Pdlim3, Pafah1b1, Ttn, Tmod1* |
| **Cell growth** | **0.014** | *Ndn, Tnn, Dclk1, Tgfb2* |
| **UHZ** |  |  |
| **Extracellular matrix organization** | **0.008** | *NPNT, COL3A1, CCDC80* |
| **LHZ** |  |  |
| **Hydrogen transport** | **1.15E-05** | *Atp6v1c1, Atp6v1a, Slc36a2, Atp6v1e1, Atp6v1b2, Atp6v1d, Atp6v0b* |
| **Skeletal system development** | **2.01E-05** | *Ibsp, Ptgs2, Col13a1, Mmp9, Dmp1, Hexb, Mmp13, Epha2, Vdr, Hoxb2, Insig2, Kazald1, Bmp8a* |
| **Vasculature development** | **2.78E-05** | *Zfand5, Nrp1, Pdgfa, Gja1, Cdh5, Cited2, Ctnnb1, Sema5a, Zfp36l1, Gpx1, Itgav, Robo4, Sox18, Lox, Flt1, Egfl7, Socs3, Tgfbr1, Myo1e, Sphk1, Fzd5, Mmp14, Kdr, Notch1, Nus1, Foxc1, Eng* |
| **Cell redox homeostasis** | **5.74E-05** | *Txnl1, Gsr, Glrx5, 2810407c02rik, Prdx4, Prdx2, Txnrd1, Pdia5, Sh3bgrl3, 2700094k13rik, Qsox1, Glrx2* |
| **Phosphate metabolic process** | **8.41E-05** | *Impa1, Prpf4b, Synj1, Aurka, Prdx2, Fes, Tgfb1, Tie1, Pak1, Csk, Map2k5, Adam10, Sgk3, Ptprg, Ndufc2, Pim1, Atp6v1h, Prkch, Ppp1cc, Atp6v1d, Atp6v1f, Map4k3, Map4k4, Hipk1, Nme1, Eif2s1, Csnk1g3, Kalrn, Ssh2, Stk17b, Abi2, Adrbk1, Cd74, Atp6v0b, Src, Atp6v0c, Gpd1l, Atp6v0e, Sbk1, Tek, Bub1, Camk2b, Flt1, Nlk, Tgfbr1, Pdk3, Raf1, Ptpn13, Ptpn12, Cdc25b, Kdr, Ccnb1, Ptp4a3, Dusp1, Csnk1d, Plk1, Atp6v1e1, Prkar1a, Jak1, Ptpn1, Abl2, F2r* |
| **Ossification** | **5.62E-04** | *Ibsp, Ptgs2, Col13a1, Kazald1, Dmp1, Mmp13, Bmp8a* |
| **Wnt receptor signaling pathway** | **0.001** | *Nlk, Tle3, Ctnnd1, Fzd5, Tax1bp3, Tcf7l2, Porcn, Ctnnb1, Sdc1, Wisp1, Macf1, Csnk1d, Sfrp4, Wif1, Csnk1g3* |
| **Proteolysis** | **0.002** | *MKRN1, ERMP1, ADAM10, PRTN3, SOCS3, MMP9, MMP16, UBE2C, MMP13, MARCH3, MMP10, CTSK, ADAMTS9, CTSE, PRCP, CFH, PHEX, ADAM12, PCSK6, CTSH, SRGN* |
| **Regulation of apoptosis** | **0.009** | *Bmi1, Mcl1, Stk17b, Prdx2, Cd24a, Cd74, Src, Tgfb1, Cited2, Timp1, Serinc3, Gpx1, Bok, Ppp2cb, Trp53inp1, Skil, Angptl4, Spp1, Tm2d1, Sgk3, Tgfbr1, Sphk1, Pim1, Birc5, Bad, Nras, Cdkn1a, Notch1, Nupr1, Hipk1, Btg2, Btg1, Psme3, Foxc1, Id3, Cln8, F2r* |
