# Supplementary material for: Synergistic co-regulation and competition by a SOX9-GLI-FOXA phasic transcriptional network coordinate chondrocyte differentiation transitions
Source: PLoS Genet. 2018 Apr 16;14(4):e1007346. doi: 10.1371/journal.pgen.1007346 (PMC5919691; doi:10.1371/journal.pgen.1007346)
Supplement: S4 Table — Genes involved in IHH, IGF, WNT, BMP, FGF, HIPPO and NOTCH signaling pathways are listed. Fisher’s exact probability test was performed by categorizing the genes into the 2 groups (PZ and PHZ versus UHZ and LHZ). The whole set of the DEGs in S2 Table was set as the background group. (DOCX) [file pgen.1007346.s008.docx]

| **Table S4 Enriched Signaling Pathways in the growth plate.** | | | | | | | |
| --- | --- | --- | --- | --- | --- | --- | --- |
| **Gene** | **H** | **M** | **Zone** | **Gene** | **H** | **M** | **Zone** |
| **Hedgehog (P-Value = 0.0034)** | | | | **Wnt (P-Value = 1.0000)** | | | |
| *Ptch1* | **1** | **1** | PZ | *Sfrp2* | **-** | **1** | PZ |
| *Gas1* | **-** | **1** | PZ | *Fzd1* | **-** | **1** | PZ |
| *Shox2* | **-** | **1** | PZ | *Dixdc1* | **-** | **-** | PZ |
| *Gli2* | **-** | **1** | PZ | *Fzd2* | **1** | **1** | PZ |
| *Cdon* | **-** | **1** | PZ | *Sost* | **1** | **1** | PZ |
| *Boc* | **-** | **1** | PZ | *Fzd7* | **-** | **1** | PZ |
| *Gli1* | **-** | **1** | PZ | *Ror2* | **1** | **1** | PZ |
| *Tulp3* | **-** | **1** | PZ | *Fzd8* | **-** | **-** | PZ |
| *Ift172* | **1** | **1** | PZ | *Sfrp1* | **-** | **1** | PZ |
| *Prrx2* | **-** | **1** | PHZ | *Fzd9* | **-** | **1** | PZ |
| *Ihh* | **1** | **1** | PHZ | *Dkk3* | **-** | **-** | PHZ |
| *Hhip* | **-** | **-** | PHZ | *Nxn* | **-** | **1** | PHZ |
| *Ndst1* | **-** | **1** | LHZ | *Wnt4* | **1** | **1** | PHZ |
| **Igf (P-Value = 0.0062)** | | | | *Wnt5b* | **1** | **1** | PHZ |
| *Igf2* | **-** | **1** | PZ | *Tcf7l2* | **-** | **1** | PHZ |
| *Igfbp7* | **-** | **-** | PZ | *Wnt5a* | **1** | **1** | UHZ |
| *Igfbp4* | **-** | **-** | PZ | *Ctnnb1* | **1** | **1** | LHZ |
| *Igfbp5* | **-** | **-** | PZ | *Csnk1d* | **-** | **-** | LHZ |
| *Grb10* | **-** | **-** | PZ | *Wisp1* | **-** | **-** | LHZ |
| *Igf2r* | **-** | **1** | PZ | *Sfrp4* | **-** | **-** | LHZ |
| *Igfbp6* | **-** | **-** | PZ | *Mbd2* | **-** | **-** | LHZ |
| *Ghr* | **-** | **1** | PZ | *Macf1* | **-** | **-** | LHZ |
| *Igf1r* | **-** | **1** | PZ | *Lrp4* | **1** | **1** | LHZ |
| *Igf1* | **-** | **1** | PZ | *Wif1* | **-** | **1** | LHZ |
| *Cilp* | **1** | **-** | PHZ | *Dkk1* | **1** | **1** | LHZ |
| *Igfbp2* | **-** | **0** | LHZ | *Nlk* | **-** | **-** | LHZ |
| **BMP, TGFβ (P-Value = 0.6060)**  **4)** | | | | *Fzd5* | **-** | **-** | LHZ |
| *Gpc3* | **-** | **1** | PZ | *Porcn* | **-** | **1** | LHZ |
| *Acvr2a* | **-** | **1** | PZ | *Ctnnd1* | **-** | **-** | LHZ |
| *Fst* | **-** | **1** | PZ | *Zeb2* | **-** | **-** | LHZ |
| *Ltbp1* | **-** | **1** | PZ | **Fgf (P-Value = 0.2497)** | | | |
| *Tgfbr3* | **-** | **1** | PZ | *Fgfr2* | **1** | **1** | PZ |
| *Tgfb3* | **-** | **1** | PZ | *Fgfrl1* | **-** | **1** | PZ |
| *Thbs1* | **-** | **1** | PHZ | *Fgfr3* | **1** | **1** | PHZ |
| *Tgfb2* | **1** | **1** | PHZ | **Hippo (P-Value = 1.0000)** | | | |
| *Bmper* | **1** | **1** | PHZ | *Fat4* | **-** | **1** | PZ |
| *Bmp6* | **-** | **1** | UHZ | *Lats2* | **-** | **-** | PHZ |
| *Eng* | **-** | **-** | LHZ | *Fat3* | **-** | **-** | LHZ |
| *Bmp2* | **1** | **1** | LHZ | *Tead2* | **-** | **1** | LHZ |
| *Smad6* | **-** | **-** | LHZ | **Notch (P-Value = 1.0000)** | | | |
| *Tgfbr1* | **1** | **-** | LHZ | *Notch3* | **-** | **1** | PZ |
| *Tgfb1* | **1** | **1** | LHZ | *Notch1* | **-** | **1** | LHZ |

Fisher’s exact probability test was performed by categorizing the genes into the 2 groups (PZ and PHZ versus UHZ and LHZ). The whole set of the DEGs in Table S2 was set as the background group.
