# Supplementary material for: Synergistic co-regulation and competition by a SOX9-GLI-FOXA phasic transcriptional network coordinate chondrocyte differentiation transitions
Source: PLoS Genet. 2018 Apr 16;14(4):e1007346. doi: 10.1371/journal.pgen.1007346 (PMC5919691; doi:10.1371/journal.pgen.1007346)
Supplement: S5 Table — The association of these TFs with human skeletal diseases and mouse skeletal phenotypes according to the OMIM and MGI databases are also summarized respectively. CSD: coefficient of standard deviation (mean normalized standard deviation); Exp: expression level; H: human skeletal disorders; M: mouse skeletal phenotypes; 1: mutations of the gene are associated with H or M; 0: mutations of the gene are not associated with H or M; -: mutations of the gene have not been reported to be associated with H or M. (DOCX) [file pgen.1007346.s009.docx]

| **Table S5 Differentially expressed transcription factors and their associations with skeletal disorders in human (H) and mouse (M)** | | | | | | | | | |
| --- | --- | --- | --- | --- | --- | --- | --- | --- | --- |
| **PZ** | **CSD** | **Exp.** | **H** | **M** | **pHZ** | **CSD** | **Exp.** | **H** | **M** |
| *Sox5* | 0.88 | 622 | **-** | **1** | *Sox9* | 0.63 | 1545 | **1** | **1** |
| *Prickle1* | 0.72 | 750 | **0** | **-** | *Tcfl5* | 0.44 | 1266 | **-** | **-** |
| *Plagl1* | 0.58 | 3988 | **-** | **1** | *Meox2* | 0.42 | 652 | **-** | **1** |
| *Gli1* | 0.53 | 1164 | **-** | **1** | *Foxa2* | 0.38 | 287 | **-** | **1** |
| *Aff3* | 0.49 | 1957 | **1** | **1** | *Trps1* | 0.36 | 2917 | **1** | **1** |
| *Creb3l2* | 0.44 | 875 | **-** | **1** | *Eya1* | 0.33 | 903 | **0** | **1** |
| *Pitx1* | 0.43 | 548 | **1** | **1** | *Rora* | 0.29 | 1513 | **-** | **-** |
| *Bnc2* | 0.40 | 1424 | **-** | **1** | *Mef2c* | 0.28 | 2894 | **0** | **1** |
| *Foxp2* | 0.39 | 913 | **0** | **1** | *Pawr* | 0.27 | 725 | **-** | **-** |
| *Nfia* | 0.39 | 709 | **-** | **1** | *Foxo1* | 0.20 | 966 | **-** | **1** |
| *Runx3* | 0.39 | 1576 | **-** | **1** | **UHZ** | **CSD** | **Exp.** | **H** | **M** |
| *Ndn* | 0.38 | 2521 | **0** | **-** | *Fos* | 0.30 | 3868 | **1** | **1** |
| *Zbtb20* | 0.37 | 1257 | **-** | **1** | *Tcf7l2* | 0.24 | 538 | **-** | **1** |
| *Hoxc8* | 0.35 | 879 | **-** | **1** | *Prrx2* | 0.22 | 757 | **-** | **1** |
| *Dbp* | 0.32 | 642 | **-** | **-** | *Bach2* | 0.19 | 284 | **-** | **-** |
| *Zfp521* | 0.29 | 1715 | **-** | **1** | **LHZ** | **CSD** | **Exp.** | **H** | **M** |
| *Hoxc10* | 0.28 | 1714 | **-** | **1** | *Irx3* | 0.82 | 1155 | **-** | **1** |
| *Shox2* | 0.28 | 632 | **-** | **1** | *Egr3* | 0.75 | 630 | **-** | **1** |
| *Hoxa9* | 0.27 | 2681 | **-** | **1** | *Irx5* | 0.54 | 977 | **1** | **1** |
| *Hoxa10* | 0.26 | 629 | **-** | **1** | *Vdr* | 0.41 | 893 | **0** | **1** |
| *Nfix* | 0.26 | 2917 | **1** | **1** | *Cebpb* | 0.38 | 1321 | **-** | **1** |
| *Wwtr1* | 0.25 | 940 | **-** | **-** | *Zfp711* | 0.37 | 553 | **-** | **-** |
| *Med12* | 0.25 | 1371 | **-** | **1** | *Foxc1* | 0.33 | 532 | **1** | **1** |
| *Gli2* | 0.24 | 716 | **0** | **1** | *Baz1a* | 0.32 | 935 | **-** | **-** |
| *Nfatc4* | 0.24 | 1368 | **-** | **1** | *Smad6* | 0.29 | 510 | **-** | **1** |
| *Lmcd1* | 0.24 | 680 | **-** | **-** | *Sox18* | 0.29 | 1091 | **0** | **-** |
| *Tsc22d3* | 0.24 | 1719 | **-** | **-** | *Nsbp1* | 0.29 | 766 | **-** | **-** |
| *Nfat5* | 0.23 | 1459 | **-** | **-** | *Ets1* | 0.29 | 2413 | **-** | **-** |
| *Hmg20a* | 0.22 | 1376 | **-** | **-** | *Sin3b* | 0.28 | 2118 | **-** | **1** |
| *Nr3c1* | 0.22 | 1250 | **0** | **-** | *Id3* | 0.28 | 4565 | **-** | **-** |
| *Tef* | 0.22 | 1832 | **-** | **-** | *Cited2* | 0.28 | 843 | **-** | **1** |
| *Smarcd3* | 0.22 | 998 | **-** | **-** | *Asf1b* | 0.24 | 717 | **-** | **-** |
| *Prdm5* | 0.21 | 1082 | **-** | **-** | *Ctnnb1* | 0.23 | 725 | **0** | **1** |
| *Nfic* | 0.21 | 4368 | **-** | **1** | *Creg1* | 0.23 | 1576 | **-** | **-** |
| *Sox6* | 0.21 | 573 | **-** | **1** | *Rb1* | 0.21 | 919 | **0** | **1** |
| *Srebf1* | 0.21 | 2024 | **-** | **-** | *Atf6* | 0.21 | 803 | **-** | **-** |
| *Lmo4* | 0.20 | 2039 | **-** | **1** | *Mafb* | 0.21 | 2632 | **-** | **1** |
| *Rere* | 0.18 | 736 | **-** | **1** | *Hmgb2* | 0.20 | 1072 | **-** | **0** |
| *Tbx15* | 0.17 | 1043 | **1** | **1** | *Nfkbia* | 0.15 | 6374 | **0** | **-** |

The association of these TFs with human skeletal diseases and mouse skeletal phenotypes according to the OMIM and MGI databases are also summarized respectively. CSD: coefficient of standard deviation (mean normalized standard deviation); Exp: expression level; H: human skeletal disorders; M: mouse skeletal phenotypes; 1: mutations of the gene are associated with H or M; 0: mutations of the gene are not associated with H or M; -: mutations of the gene have not been reported to be associated with H or M.
