# Supplementary material for: Synergistic co-regulation and competition by a SOX9-GLI-FOXA phasic transcriptional network coordinate chondrocyte differentiation transitions
Source: PLoS Genet. 2018 Apr 16;14(4):e1007346. doi: 10.1371/journal.pgen.1007346 (PMC5919691; doi:10.1371/journal.pgen.1007346)
Supplement: S6 Table — We defined the spacer length Ln as the number of DNA bases located between the paired SOX9 core binding consensus, represented as 5’-AACAA(Ln)-TTGTT3’. (DOCX) [file pgen.1007346.s010.docx]

| **Table S6 Enriched SOX9 dimer motif in PZ and PHZ** | |
| --- | --- |
| **PZ** | |
| W=20, P=4.3e-7, L=4-bp | W=28, P=1.2e-14, L=8-bp |
| 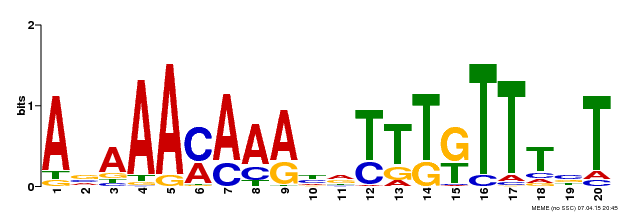 | 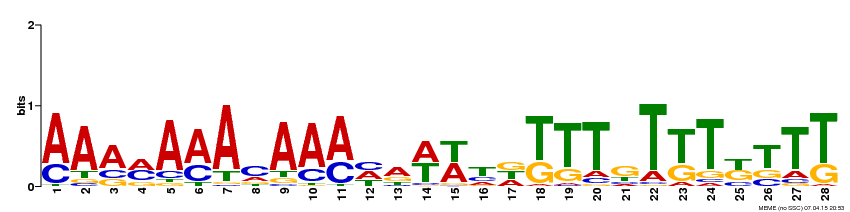 |
| W=21, P=1.5e-9, L=10-bp | W=29, P= 8.3e-7, L=8-bp |
| 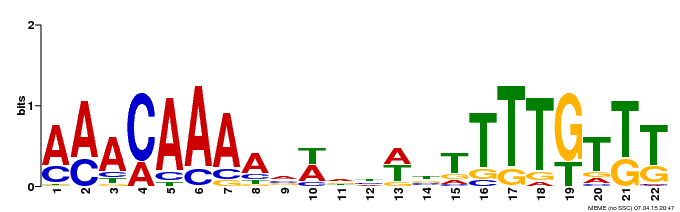 | 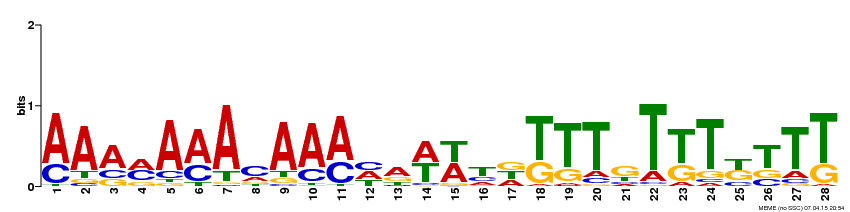 |
| W=22, P=4.0e-12, L=13-bp | W=30, P=8.3e-7, L=10-bp |
| 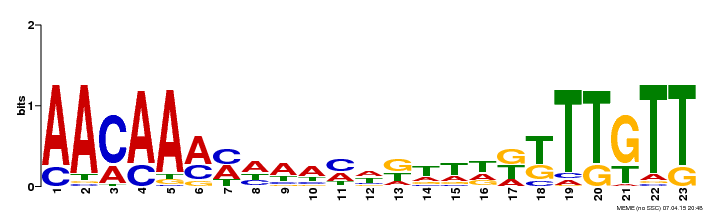 | 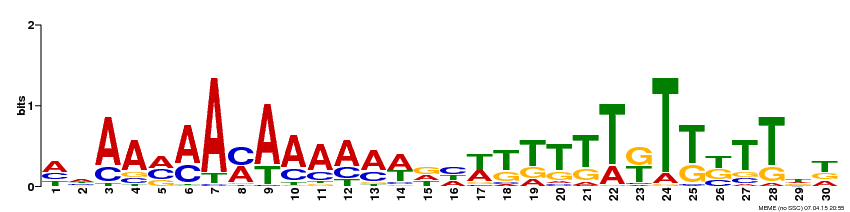 |
| W=23, P=4.0e-12, L=11-bp | W=31, P=9.1e-6, L=10-bp |
| 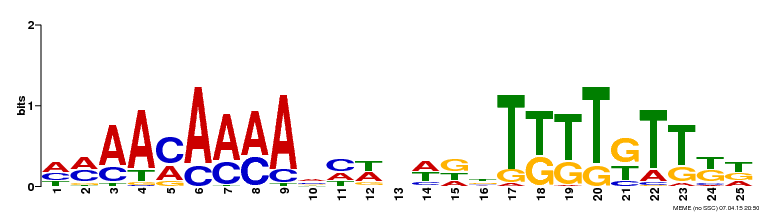 | 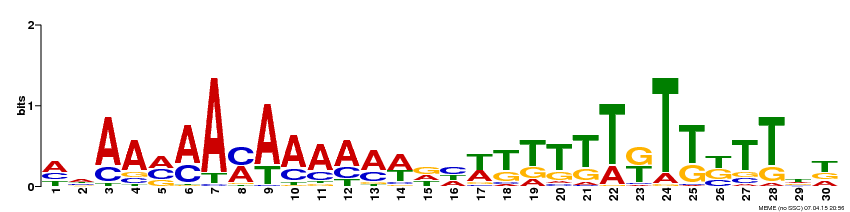 |
| W=24, P=1.3e-7, L=11-bp | W=32, P=1.2e-6, L=13-bp |
| 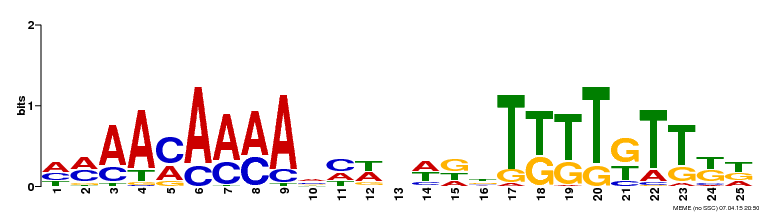 | 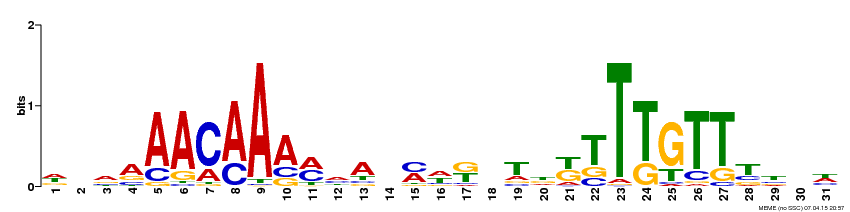 |
| W=25, P=2.8e-13, L=4 or 12-bp | W=33 - 36 |
| 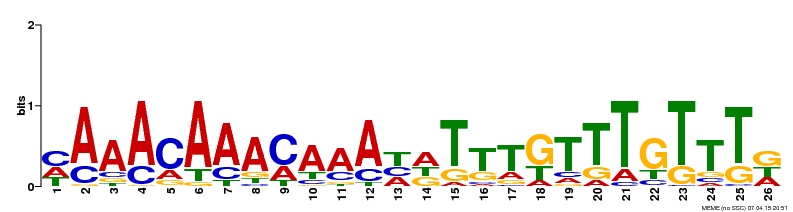 | N.S. |
| W=26, P=2.8e-13, L=4 or 12-bp | W=37, P=1.1e-6, L=9-bp |
| 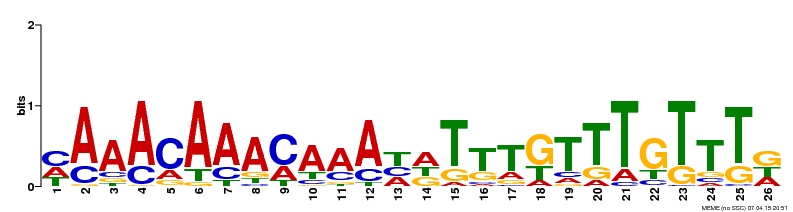 | 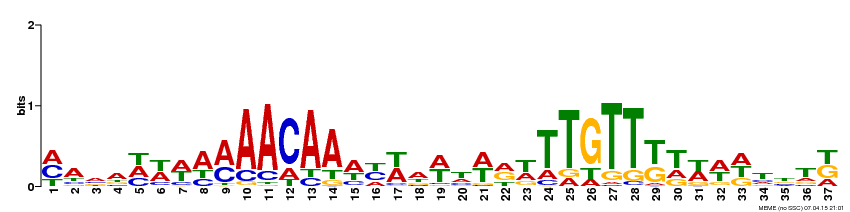 |
| W=27, P=1.2e-13, L=4 or 12-bp |  |
| 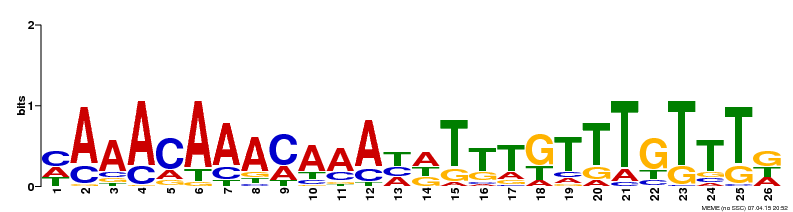 |  |
| **PHZ** | |
| W=21 - 28 | W=30, P=2.6e-6, L=4, 10 or 16-bp |
| N.S. | 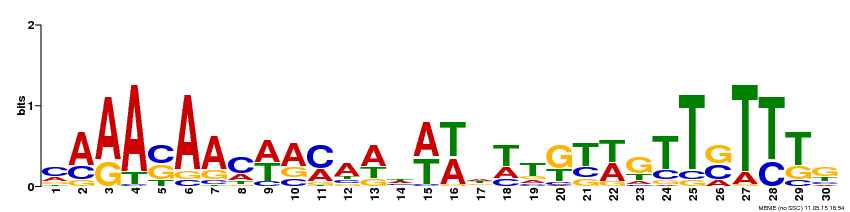 |
| W=29, P= 2.6e-6, L=4, 10 or 16-bp | W=31 - 37 |
| 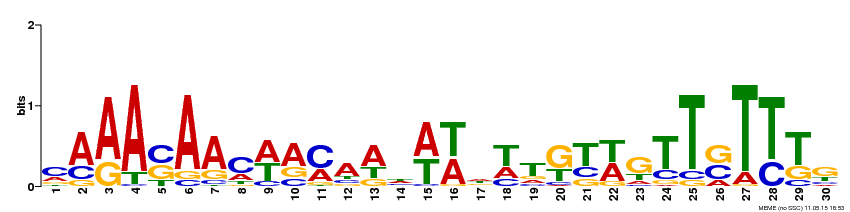 | N.S. |

**W: Width of dimer motif; P: P-Value; L: Length of spacer; N.S.: Statistically Not Significant**
