# Supplementary material for: Synergistic co-regulation and competition by a SOX9-GLI-FOXA phasic transcriptional network coordinate chondrocyte differentiation transitions
Source: PLoS Genet. 2018 Apr 16;14(4):e1007346. doi: 10.1371/journal.pgen.1007346 (PMC5919691; doi:10.1371/journal.pgen.1007346)
Supplement: S9 Table — Conserved SOX9 binding sites in the regulatory elements of Cyr61, Trps1 and Ptch1 were selected for ChIP-PCR validation. The genomic coordinates of these SOX9 binding site and their relative position to the nearest SOX9, GLI1 and GL3 binding peaks were shown. (DOCX) [file pgen.1007346.s013.docx]

| Table S9 SOX9 binding motifs identified and validated in *Cyr61*, *Trps1* and *Ptch1* genes. | | | | | | | |
| --- | --- | --- | --- | --- | --- | --- | --- |
| **Genes** | | **Motif loci** | **Type of binding sites** | **Distance to TSS** | **Distance to ChIP-seq peaks** | | |
|  |  |  |  |  | **GLI1** | **GLI3** | **SOX9** |
| *Cyr61* | | chr3: 145313558 | Sox9_COL2C2 | 606 | none | -155 | 223 |
| *Trps1* | | chr15: 50719966 | Sox9_COL2C2 | -1621 | 2208 | 107 | 116 |
| *Ptch1* | I | chr13: 63667875 | Sox9_COL2C3 | -6599 | 69 | 69 | 157 |
|  | II | chr13: 63668620 | Sox9_COL2C2 | -5854 | -814 | 149 | -1234 |
